# Characterization of a functional endothelial super-enhancer that regulates ADAMTS18 and angiogenesis

**DOI:** 10.1093/nar/gkab633

**Published:** 2021-07-28

**Authors:** Isidore Mushimiyimana, Henri Niskanen, Mustafa Beter, Johanna P Laakkonen, Minna U Kaikkonen, Seppo Ylä-Herttuala, Nihay Laham-Karam

**Affiliations:** A. I. Virtanen Institute for Molecular Sciences; University of Eastern Finland; Kuopio 70211, Finland; A. I. Virtanen Institute for Molecular Sciences; University of Eastern Finland; Kuopio 70211, Finland; A. I. Virtanen Institute for Molecular Sciences; University of Eastern Finland; Kuopio 70211, Finland; A. I. Virtanen Institute for Molecular Sciences; University of Eastern Finland; Kuopio 70211, Finland; A. I. Virtanen Institute for Molecular Sciences; University of Eastern Finland; Kuopio 70211, Finland; A. I. Virtanen Institute for Molecular Sciences; University of Eastern Finland; Kuopio 70211, Finland; Heart Center and Gene Therapy Unit; Kuopio University Hospital; Kuopio 70029, Finland; A. I. Virtanen Institute for Molecular Sciences; University of Eastern Finland; Kuopio 70211, Finland

## Abstract

Super-enhancers are clusters of enhancers associated with cell lineage. They can be powerful gene-regulators and may be useful in cell-type specific viral-vector development. Here, we have screened for endothelial super-enhancers and identified an enhancer from within a cluster that conferred 5–70-fold increase in transgene expression. Importantly, CRISPR/Cas9 deletion of enhancers demonstrated regulation of *ADAMTS18*, corresponding to evidence of chromatin contacts between these genomic regions. Cell division-related pathways were primarily affected by the enhancer deletions, which correlated with significant reduction in cell proliferation. Furthermore, we observed changes in angiogenesis-related genes consistent with the endothelial specificity of this SE. Indeed, deletion of the enhancers affected tube formation, resulting in reduced or shortened sprouts. The super-enhancer angiogenic role is at least partly due to its regulation of *ADAMTS18*, as siRNA knockdown of *ADAMTS18* resulted in significantly shortened endothelial sprouts. Hence, functional characterization of a novel endothelial super-enhancer has revealed substantial downstream effects from single enhancer deletions and led to the discovery of the *cis*-target gene *ADAMTS18* and its role in endothelial function.

## INTRODUCTION

Vascular endothelium plays a critical role in the regulation of vascular function and homeostasis (1). Major cardiovascular diseases, including ischemia and coronary artery disease are associated with impaired endothelial function (1,2); subsequently, the endothelium has been a therapeutic target for many cardiovascular complications (2,3). Cardiovascular gene therapy represents the fourth most popular gene therapy application, representing 7.4% of performed clinical trials (4). Most of these trials have been focusing on therapeutic angiogenesis to stimulate and increase the growth of new blood vessels from pre-existing vessels to recover blood flow to ischemic tissues (5). In clinical trials, various growth factors have been harnessed to stimulate the growth of vasculature; however, very few results with clinical significance have been achieved (6). Some of the issues linked with failed therapeutic angiogenesis have included inefficient gene transfer, short term duration of transgene expression as well as challenges related to the selection of study endpoint (6,7). In addition, although viral vectors are regarded as the most efficient gene transfer vectors due to their combined ability of high transduction and the capacity for carrying transgenes (8), lack of targeting of these to specific cells/tissue has limited their clinical applicability (9,10). As such a new avenue for inducing cell-type specificity in viral vectors is necessary. One possible way forward is to utilize genomic elements, such as enhancers, in the vectors to limit the transgene expression to specific cell-types.

Enhancers are recognized as *cis*-regulatory DNA elements that can increase expression of target genes in cooperation with promoters (11). Within an enhancer sequence there are multiple transcription factor binding sites that are required for the regulation of enhancer activity (12). Active enhancers in the context of the genome are primarily predicted in accessible chromatin regions based on a combination of specific features, including deposition of particular histone marks such as the monomethylated histone 3 at lysine 4 (H3K4me1) and acetylated H3K27 (H3K27ac); enhancer RNA (eRNA) production; as well as evidence of coactivator protein p300 binding (13–16). Sequence variants in enhancers have been associated with disease (17,18) and manipulation of these in animal studies have impacted phenotype (19). In addition to constitutive enhancers, recently a concept of super-enhancers (SE) has been used to describe a cluster of enhancers that span a genomic locus, densely bound by master regulators and coactivators (20). SE were demonstrated to exhibit high cell-type-specificity and to control the expression of genes essential for lineage specificity (20–22) and pluripotency (23,24) as well as driving biogenesis of miRNA network essential for cell identity (25). In addition, genes associated with SE generally present higher expression levels compared to genes associated with typical enhancers (20). Thus, SE can be exploited to induce robust, cell-type specific gene expression in the context of gene therapy vectors.

In this study, we identified and functionally validated an endothelial specific SE (SE12313). We utilized an enhancer (E2) from this SE to induce endothelial specific gene expression with a lentiviral vector. We also demonstrated that E2 contains a HIF1α binding site and hypoxic conditions induced further activation of the reporter expression. Genomic deletion of this enhancer using CRISPR/Cas9 and genome-wide sequencing studies identified possible SE12313 targets, which included *ADAMTS18*, and the associated biological processes, including cell division. Importantly, functional analysis of the enhancer deleted cells showed a detrimental effect on tube formation. This effect is likely due to SE12313 regulation of *ADAMTS18, since* knockdown of *ADAMTS18* expression also reduced endothelial cell sprouting, which demonstrates a novel and previously uncharacterized function for *ADAMTS18*. Our data present the first evidence of an important role for SE12313 and *ADAMTS18* in endothelial function.

## MATERIALS AND METHODS

### Vector construction

Candidate enhancers from SE and gene promoters were amplified from human umbilical vein endothelial cells (HUVECs) genomic DNA using Phusion Hot Start II DNA Polymerase (Thermo Fisher Scientific) and specific primers (Supplementary Table S1) which were then cloned into a blunt cloning plasmid (pCR-blunt; Zero blunt PCR™ cloning kit; TheromFisher) and their sequence confirmed by Sanger sequencing (Macrogen Europe). A lentiviral vector plasmid (pLV-minP) was generated containing a minimal promoter (minP) driving the expression of enhanced GFP by replacing the *PGK* (Phosphoglycerate kinase 1) promoter in pLV1-GFP (26) with a double stranded linker expressing the minP (Supplementary Table S1). Likewise, TK minimal (TK; Thymidine kinase), *ADAMTS18 (*A Disintegrin-Like And Metalloprotease With Thrombospondin Type 1 Motif, 18) and *NUDT7* (Nudix Hydrolase 7) promoters replaced the PGK promoter in pLV1-GFP to generate pLV-TK-GFP, pLV-ADAMTSp-GFP and pLV-NudT7p-GFP, respectively. Selected enhancer regions were subcloned into pLV plasmids from pCR-blunt using standard restriction cloning method. Firefly luciferase pLV construct was generated by replacing GFP in pLV-minP-GFP with Luciferase (Luc) reporter gene to make pLV-minP-Luc vector construct.

### Cell lines, cell culture and transfections

Human cell lines A549 (ATCC: CCL-185), 293T (ATCC: CRL-1573), HeLa (ATCC: CRM-CCL-2, HepG2: (ATCC: HB-8065), EA.hy 926 (ATCC: CRL-2922), and mouse cell lines MOVAS (ATCC: CRL-2797) and MS1 (ATCC: CRL-2279) were maintained in Dulbecco's Modified Eagle's Medium (DMEM)-high glucose (Sigma) supplemented with 10% FBS and 100 mg/ml Penicillin and 100 U/ml of Streptomycin. HaSMC (ATCC: PCS-100-012) were grown in complete Vascular smooth muscle cell media (ATCC). Primary endothelial cells (ECs), HUVECs were isolated by collagenase digestion (27) from human umbilical cords obtained from the maternity ward of the Kuopio University Hospital (KUH), with the approval from the Research Ethics Committee of the Northern Savo Hospital District (341/2015). Pig EC were a kind gift from Dr O-P Hätinen (28). Primary EC at early passage were maintained in Endothelial Cell Growth Basal Medium (EBM; Lonza) supplemented with endothelial growth medium SingleQuotes (thereafter referred to as EGM; Lonza) in cell culture flasks coated with 10 μg/ml fibronectin and 0.05% gelatin (Sigma). Hypoxia induction was achieved by incubation of the cells in Ruskinn InVivo2 400 hypoxia incubator at 1% O_2_ and 5% CO_2_.

Dicer-substrate oligonucleotides (DsiRNA; IDTDNA) were transfected into EC using Oligofectamine (Invitrogen) according to the manufacturer's instruction. For functional assays, DsiRNA-treated cell were used 1 day after transfection, for gene expression analysis they were analysed also after 48h of transfection.

### Lentiviral production and transductions

Viral like particles (VLPs) encapsulating pLV-(Ex) enhancer constructs or control vector without an enhancer, pLV-minP were produced in 293T cells by cotransfection with the packaging constructs pVSV-g (29), pREV (30) and pMDg (31), using calcium phosphate method as previously described (32). Empty LV were produced by transfection of only the packaging plasmids (pVSV-g, pREV, pMDg) to produce particles devoid of the vector RNA genome.

To perform transductions, cells were seeded in 6-well cell culture plates at a density of 5 × 10^4^ per well. The following day, the cells were transduced with medium containing VLPs in duplicate wells for each vector. Empty LV and LV with matching promoter but without enhancer were the control transduction in each experiment. Transducing medium was replaced with 2 ml of fresh culture medium after 5h and incubated for 3 days at 37°C and 5% CO_2_. The cells were thereafter collected by trypsinization and fixed with 2% formaldehyde (in 1XPBS; Sigma).

### Flow cytometry and luciferase reporter assay

Flow cytometric analysis of GFP expression in transduced cells was performed using FACS Calibur with a 488-nm laser (Becton Dickinson, NJ). GFP positive marker was set using cells transduced with VLPs devoid of packaged viral vector RNA to exclude autofluorescence.

Luciferase activity was assessed using luciferase reporter assay kit according to the manufacturer's instructions (E1910, Promega). Transduced cells were seeded into Tissue Culture Treated B&W Isoplate-96 (PerkinElemer) at a density of 1.2 × 10^4^ cells/well and incubated for 48 h. Luciferase activity was measured with CLARIOstar microplate reader (BMG LABTECH; λ = 562 nm) and normalized to the signal obtained with LV-minP vector without enhancer.

### Cell proliferation, cell cycle analysis and angiogenesis assays

Cell proliferation was performed using xCELLigence Real-Time Cell Analyzer Multi-Plate (RTCA MP) instrument (ACEA; Biosciences). Background impedance was determined using 100 μl of culture medium prior to cell seeding into an E-plate 16 PET (ACEA; Biosciences). Subsequently, cells were added to duplicate wells of the plate at a density of 5 × 10^3^ per well and the plate was incubated in RTCA MP. Cell proliferation was monitored every 2h for a total of 52h.

Cell cycle analysis was performed using NucleoCounter NC3000™ (Chemometec) according to the manufacturer's instructions. Cells were seeded in duplicate wells the day before at 2.5 × 10^5^ cells/well in six-well plates. The cell lysate was loaded on a NC-slide A2™ (Chemometec) and analysed in NucleoCounter NC3000 using the 2-Step Cell Cycle Assay program.

Angiogenesis bead assay (33) was performed as previously described (34). HUVECs were seeded on cytodex microcarrier beads (GE Healthcare, Little Chalfont, UK) and embedded in a fibrin gel. Human lung pulmonary fibroblasts were cultured on top of the gels. On day 3, wells were fixed, permeabilized and stained with phalloidin-A635 (F-actin; red) and DAPI (blue). Imaging was performed using Zeiss LSM800 confocal laser scanning microscope (405/555nm diode lasers, 10x/0.3 PlanApo objective, 512 × 512 frame size). Image processing and quantitative analysis for 20–23 beads per group was performed using ImageJ Fiji (35,36).

*In vitro* tube formation assay was performed by plating EA.hy 926 clones of ΔE2 and ΔSE12313-b and EA.hy926 control cell line onto a layer of growth factor reduced matrigel (Corning, Inc., New York, NY) in 48-well plate at a density of 6 × 10^4^ cells/well. The cells were subsequently incubated at 37°C and live imaged with IncuCyte S3 Live-Cell Analysis System (Sartorius, Göttingen, Germany) using a 4× objective lens. All images taken after 16h of incubation were analyzed for tube formation with Image J Angiogenesis Analyzer software.

### CRISPR/Cas9-mediated deletion of enhancers

CRISPR/Cas9-mediated deletion of constituent enhancers were performed using Cas9/RNA ribonucleoprotein (RNP) based approach (Alt-R CRISPR-Cas9 system; IDTDNA). Briefly, guide RNA sequences flanking target enhancer deletions were designed using CRISPR tool (37) and ordered as crRNAs (IDTDNA; Supplementary Table S2). These crRNAs were subsequently annealed to tracrRNA and mixed with recombinant Cas9 protein (HiFi Cas9 Nuclease V3; IDTDNA) to form RNP complexes. The RNP complexes were then reverse transfected into cells (4 × 10^5^ EA.hy926 cells/well were plated into 96-well plate) using Lipofectamine CRISPRMAX reagents (Thermo Fisher Scientific). The cells were collected 48h post transfection and diluted for clone selection. The culture medium was refreshed every 4 days until single discrete clones from single cells were clearly visible and genotyped by PCR.

### Nucleic acid extraction and amplification

Genomic DNA was extracted from cells using NucleoSpin tissue kit (Macherey-Nagel) according to the manufacturer's instructions. 50–100 ng of gDNA was used for PCR using specific primers flanking target deletion sites (Supplementary Table S2) and Phire Hot Start II DNA Polymerase (Thermo Fisher Scientific). PCR products were then analyzed by electrophoresis in 1% agarose gel.

Total RNA isolation was performed using RNeasy Mini kit (QIAGEN). One μg of RNA was subjected to DNase treatment with TURBO DNA-free Kit (Invitrogen) followed by cDNA synthesis with reverse transcription (RT) using RevertAid First Strand cDNA synthesis Kit (Thermo Fisher Scientific). mRNA expression was quantitated in cDNA samples using real time quantitative PCR (qPCR), with 2x Universal PCR Master mix (Thermo Fisher Scientific), and specific primer Taqman-based probe assays (Supplementary Table S3). Gene expression of interest was standardized to a housekeeping gene (*ACTB*) and the relative expression was calculated using the ΔΔCT method (38).

### RNA library preparation

DNase-treated RNA samples were checked for integrity on Fragment Analyzer (Agilent) using RNA Kit (15NT; DNF-471). RNA library preparation was performed using NEBNext Ultra II Directional RNA Library Prep Kit for Illumina (E7765; New England BioLabs) according to the manufacturer instructions. Briefly, 0.5 μg of each RNA sample was taken for ribosomal RNA depletion followed by fragmentation, cDNA synthesis and library enrichment with 11 cycle of PCR amplification. RNA-seq libraries were then quantified using Qubit dsDNA HS Assay Kit (ThermoFisher Scientific) and quality checked on Agilent 2100 Bioanalyzer using Agilent DNA 100 kit. All libraries were pooled in equimolar amount (4 nM for each) and sequenced with Nextseq 550 (Illumina) using single end 75 cycles for 10 million reads per sample.

### RNA-seq, ChIP-seq, GRO-seq and Hi-C data analysis

The raw RNA sequencing reads were trimmed and filtered using Trim Galore (V.O4.4) with Phred quality score cutoff 30. Processed reads were mapped to reference human genome assembly (GRCh37/hg19) using STAR version 2.5.4b (39) with options outFilterMismatchNoverLmax 0.04 and outFilterMultimapNmax 10. Next, aligned reads mapping to features were assigned using featureCounts (Rsubread 1.32.4) with the Gencode V19 GTF. CPM <1 was used as threshold to remove transcripts with low counts and not presented in at least two libraries above this threshold. Library sizes were normalized using Trimmed Mean of M-values (TMM) and used quasi-likelihood *F*-testing to estimate differential expression using edgeR (3.24.3). Differential expressed transcripts were determined using fold change (FC) of ≥1.5 or ≤–1.5 and false discovery rate (*FDR)* <0.05. Gene ontology (GO) analysis of differential expressed genes (DEGs) was performed using Database for Annotation Visualization and Integrated Discovery (DAVID) v6.8 (40). Both upstream regulators and canonical pathways associated with DEGs were identified using IPA tool (Ingenuity Pathway Analysis; QIAGEN).

To assess transcription factors (TFs) binding to enhancer regions, we used publicly available data of TFs Chip-Seq (Supplementary Table S4), this data was processed as described in (41). Enrichment of specific TF at target enhancer was determined by the number of binding sites within an enhancer locus. Histone marks were analysed from the following GEO repository data GSE109625 and processed as described in (41). Deposits of histone marks were quantified for 50 bp bins at 3Kb region centered around the enhancer coordinates using HOMER annotatePeaks.pl. Histone marks analysis for enhancers within SE12313 and SE26147 were calculated for 2 kb regions around the center of the enhancer. Data was visualized using Rstudio and heatmap.2.

Publicly available data of GRO-seq and Hi-C from HUVEC (GSE94872) were used to assess genome-wide nascently transcribed RNA and chromatin interactions, respectively. Hi-C data from other cell lines are listed in Supplementary Table S4. The GRO-seq and Hi-C data were processed as described in (41).

### Statistical analysis

Data were statistically analyzed using Prism 9.1 (GraphPad). The data are presented as mean ± standard error of the mean (SEM), n represents the number of independent experiments unless otherwise specified. Statistical significance was ascribed for two-tailed at *P* < 0.05. Data that demonstrated homogeneous variance were tested using Student's *t*-test or multiple analysis of variance and Tukey's multiple comparison otherwise non-parametric data were tested using Kruskal Wallis analysis with Dunn's multiple comparison for three or more groups.

## RESULTS

### Identification and characterization of a functionally active endothelial enhancer

**Figure 1.**
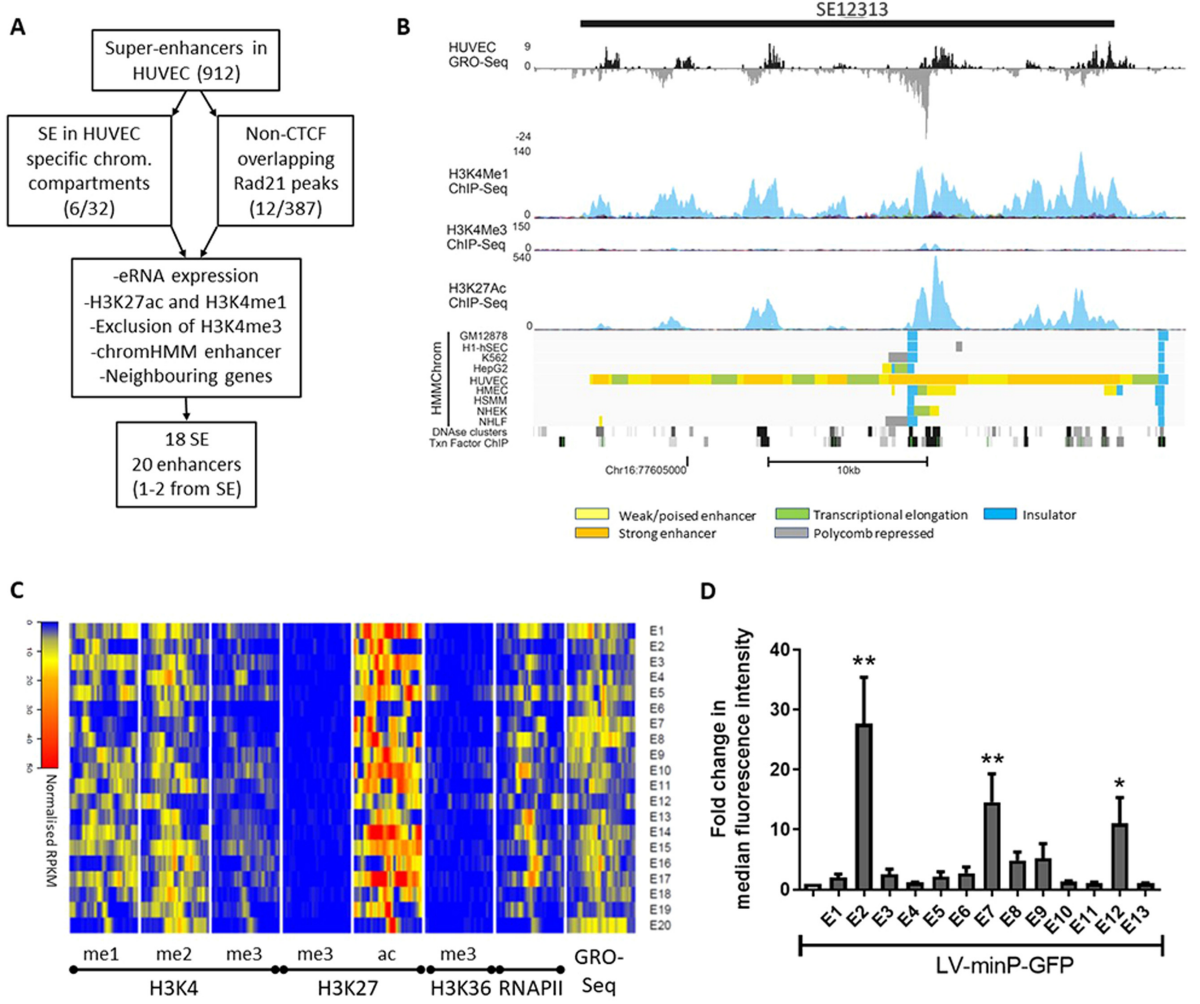
Selection criteria for endothelial specific SE and identification of functionally active enhancers. *(***A**) Schematic diagram of the selection process for endothelial specific SE. (**B**) USCS Genome browser view of a representative SE, showing tracks used for selection of the SEs. Normalized GRO-seq tags and ChIP-seq tags for H3K4me1, H3K4me3 and K3K27ac as well as DNaseI and transcription (Txn) factors ChIP clusters are shown for HUVECs. Colour key for the HMMChrom track is presented. (**C**) Heatmap of ChIP-seq data from HUVECs demonstrating deposition of specific histone marks and RNAIIP binding, as well as GRO-seq data indicating eRNA expression for the selected enhancers for a 3Kb region around the centre of enhancers. (**D**) HUVEC were transduced with LV-minP-GFP or matching vectors encoding the indicated enhancers. GFP fluorescence was quantitated using flow cytometry. Data is presented mean ± SEM (*n* = 3), statistical significance is ascribed at *P < 0.05, **P < 0.01.

To identify functionally active endothelial enhancer, first a selection strategy was applied to the previously assigned endothelial-specific SE in HUVEC (21). The starting point of this selection criteria (Figure 1A) was to limit the list of 912 SE to those either located in HUVEC-specific active chromatin compartments (*n* = 32; 41) or that overlap CTCF-independent RAD21 binding sites (*n* = 387; Figure 1A; details of data files in Supplementary Table S4). These sites are indicative of cell-type-specific regulatory elements, as CTCF-independent RAD21 binding sites show cell-type-specific regulatory interactions, binding Mediator and lineage-determining TFs (41–44). Furthermore, regions were manually selected according to the following criteria: (i) expression of eRNA in global run-on sequencing (GRO-seq) of HUVEC (45); (ii) evidence of an epigenetic signature of enhancers including presence of histone marks H3K4me1 and H3K27ac but exclusion of H3K4me3; (iii) evidence of DNase I hypersensitivity that are typical of genomic regulatory regions (14); (iv) specific assignment of enhancer in HUVEC and not in other cell-types in the chromatin state segmentation (46) and (v) consideration of neighbouring genes of interest such as those known to be important in endothelial cell biology and function (Figure 1B). From this screen 18 SE were selected and one to two individual enhancers from within the SE were chosen for cloning into lentiviral vector, favouring those with the highest H3K27ac marks or demonstrating the highest eRNA expression within the enhancer cluster (Table 1). From histone ChIP-seq and GRO-seq analysis of HUVEC, it can be seen that all the selected enhancers exhibited high eRNA expression and deposition of enhancer marks H3K4me1, H3K4me2 and H3K27ac and were devoid of repressive H3K27me3 mark and markers of promoters (H3K4me3) or gene body (H3K36me3; Figure 1C).

**Table 1.** Summary of the enhancers tested, their chromosomal location, the associated super-enhancer and nearest gene, as well as some characteristics of these enhancers

Enhancer name	Chromosome	Genome location	Start	End	Super-enhancer	Nearest gene	Size (bp)	HIF-1a peak	Sub enhancer most eRNA/H3K27ac
**E1**	chr16	q23.1	77618809	77620518	12313	NUDT7	1709	no	yes
**E2**	chr16	q23.1	77627867	77630464	12313	NUDT7	2597	yes	no
**E3**	chr1	p22.1	94557033	94558672	1436	ABCA4	1639	no	yes
**E4**	chr8	q13.2	68920525	68922184	30835	PREX2	1659	no	no
**E5**	chr1	q21.3	154456732	154458322	1941	SHE	1590	no	no
**E6**	chr18	q22.3	68703546	68705154	14735	GTSCR1	1608	no	yes
**E7**	chr5	q34	163186103	163188197	26417	MAT2B	2094	no	yes
**E8**	chr15	q14	39119680	39121858	10383	C15orf53	2178	no	yes
**E9**	chr2	q32.2	191835234	191837164	18408	STAT1	1930	no	no
**E10**	chr2	q32.2	191844637	191845874	18408	STAT1	1237	yes	yes
**E11**	chr13	q12.2–12.3	28929966	28931948	8302	FLT1	1962	no	yes
**E12**	chr13	q12.3	28966358	28969007	8308	FLT1	2649	no	yes
**E13**	chr5	q35.1	172175112	172176252	26215	DUSP1	1140	yes	yes
**E14**	chr1	q31.3	61426428	61427809	1049	NFIA-AS2	1381	no	yes
**E15**	chr10	q26.2	129774992	129776872	4695	PTPRE	1880	no	yes
**E16**	chr14	q23.1	61821708	61823382	9678	TMEM30B/PRKCH	1674	no	yes
**E17**	chr14	q23.1	62027908	62029491	9698	LOC101927780	1583	no	yes
**E18**	chr7	q31.1	107932985	107934607	29577	NRCAM	1622	no	yes
**E19**	chr8	q11.23	55248579	55250316	30727	SOX17	1737	no	yes
**E20**	chr2	q31.1	169857150	169858547	17789	ABCB11	1397	no	yes
**E1L**	chr16	q23.1	77618219	77620518	12313	NUDT7	2299	no	yes
**E2(875)**	chr16	q23.1	77628806	77629681	12313	NUDT7	875	yes	no
**E2(1284)**	chr16	q23.1	77628397	77629681	12313	NUDT7	1284	yes	no
**E2(1814)**	chr16	q23.1	77627867	77629681	12313	NUDT7	1814	yes	no
**E2(1658)**	chr16	q23.1	77628806	77630464	12313	NUDT7	1658	yes	no
**SE12313-a**	chr16	q23.1	77598373	77600312	12313	NUDT7	1939	no	no
**SE12313-b**	chr16	q23.1	77602270	77604988	12313	NUDT7	2718	no	no
**SE12313-c**	chr16	q23.1	77607759	77610243	12313	NUDT7	2484	no	no
**SE26417-a**	chr5	q34	163184684	163187040	26417	MAT2B	2356	no	yes
**SE26417-c**	chr5	q34	163193649	163195667	26417	MAT2B	2018	no	no
**SE26417-d**	chr5	q34	163201063	163202866	26417	MAT2B	1803	no	no

In the primary functional screen, following LV-E(x) transduction of HUVECs, we identified three putative endothelial enhancers (E2, E7 and E12) of varying activity (Figure 1D and Supplementary Figure S1a). Although in the primary screen we used both pLV-minP-GFP and pLV-Tk-GFP, there appeared to be no difference in the pattern of activation of these promoters by the tested enhancers (Supplementary Figure S1b), however since the minP had lower basal expression (data not shown), we continued with LV-minP-based vectors. The highest activation of the minP was achieved by E2 which demonstrated approximately a 30-fold increase in enhanced green fluorescent protein (GFP) level in HUVEC. We also tested some of these enhancers with luciferase, which is frequently used in enhancer reporter plasmids, to rule out differences in reporter sensitivities. Luciferase activity was increased by E2 and to a lesser extent E7 but not by E1, similarly to the enhancer activities seen with the GFP reporter (Supplementary Figure S1c).

**Figure 2.**
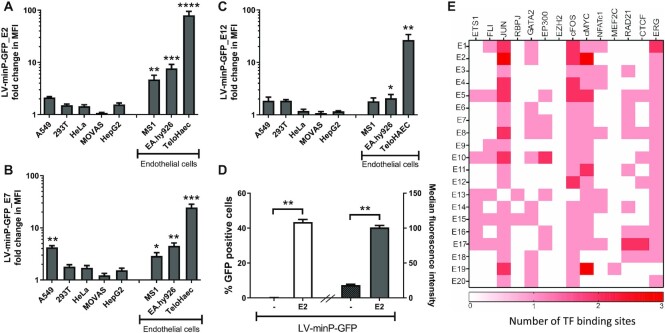
Characterization of endothelial specificity of active enhancers and comparison of their transcription factor binding. Different cell lines were transduced with control vector LV-minP-GFP and LV-minP-GFP-E2 (**A**) or -E7 (**B**) or -E12 (**C**). GFP expression was assessed with flow cytometry after 3days of transduction. Fold change in median fluorescence intensity (MFI) was calculated by comparison to LV-minP-GFP transduced cells. (**D**) Primary pig EC were transduced with LV-minP-GFP-E2 (E2) or control vector LV-minP-GFP (–) and the GFP fluorescence was quantified by flow cytometry; both the percentage of GFP positive cells (unfilled bar) and their median fluorescence intensity (filled bars) are shown. Data is presented mean ± SEM (*n* ≥ 3), *P < 0.02, **P < 0.002, ***P < 0.0005, ^****^P < 0.0001. (**E**) Heatmap of transcription factor binding peaks for all enhancers derived from ChIP-seq from HUVECs (number of binding sites is indicated by the colour gradation).

To test the endothelial specificity of the active enhancers, different cell lines were transduced with LV-E2, E7 and E12. As can be seen in Figure 2A, E2 demonstrated the best specificity, as determined by significant activation of GFP expression only in ECs, EA.hy926 and TeloHAEC, but not in other cell-types including A549, HeLa, 293T, MOVAS and HepG2. E7 was active in both endothelial and epithelial cells (Figure 2B) while E12 demonstrated activation only in some EC (Figure 2C). Importantly, E2 was active in EC of different species including mouse endothelial cell line MS1 (Figure 2A) and in primary pig ECs (pEC; Figure 2D). According to dbSuper (47), a database of super-enhancers that have been identified in 102 human and 25 mouse cell lines, SE12313 (to which E2 belongs) was only identified in EC and the U87 glioblastoma cell line. In line with this, E2 also exhibited moderate activity in U87 cells as did E7 and E12 (Supplementary Figure S1d).

We next sought to understand what confers the high EC specificity of E2. To assess this, we compared the TF binding at the enhancers using public ChIP-sequencing data from HUVECs. As can be seen in Figure 2E, E2 bound JUN, cMyc and cFOS at multiple sites, and to a lower extent also ERG and GATA2 (their location can also be found in Figure 4). However, E2 was not unique in recruiting these TFs, rather most of the enhancers tested bound JUN, cMyc, cFOS and ERG in line with these factors being important for the establishment of endothelial enhancers (21,48). Furthermore, other non-active enhancers recruited additional TFs such as ETS1. None of the enhancers tested recruited the methyltransferase EZH2 that can result in transcriptional repression, which is also consistent with the absence of H3K27me3 in these enhancers (Figure 1B).

### One enhancer from the cluster dominates in a super-enhancer

**Figure 3.**
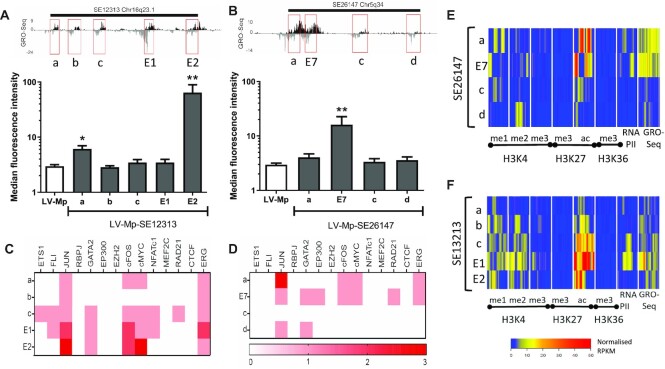
Characterization of activity, transcription factor binding and histone modifications for all enhancers within SE clusters. Examination of all enhancers within SE clusters SE12313 (**A**) and SE26147 (**B**). The regions tested are indicated in the GRO-seq track (top panels) and the activity of these regions is determined following LV transduction of HUVEC and GFP quantitation with flow cytometry (bottom graphs; data presented as mean ± SEM, *n* = 3). TF binding at enhancer regions of SE12313 (**C**) and SE26147 (**D**). The heatmap represents the number of TF binding peaks derived from ChIP-seq from HUVECs. Heatmap of Histone marks (ChIP-seq) and eRNA expression (GRO-seq) for all enhancers from SE12313 (**E**) and SE26147 (**F**) for 2kb area around the centre of the enhancer.

Interestingly, both E1 and E2 are from the same SE, SE12313, and yet only E2 demonstrated enhancer activity in our reporter systems. To investigate how individual enhancers from the same SE behave, the activity of all the individual enhancers (as indicated by evidence of eRNA transcription) from two SEs, SE12313 (Figure 3A) and SE26147 (Figure 3B) was tested. In both SE clusters, one enhancer dominated in activity over the others, which exhibited either no or moderate activity in the LV vector. E2 did not have a unique set of bound TFs in comparison to the other enhancers within SE12313 (Figure 3C), whereas E7 did recruit the most TFs compared to the remaining enhancers within SE26147 (Figure 3D). Likewise, the density of the histone marks in the active enhancers were either lower (E2; Figure 3E) or equal to (E7; Figure 3F) but not greater than the other enhancers within the SE.

**Figure 4.**
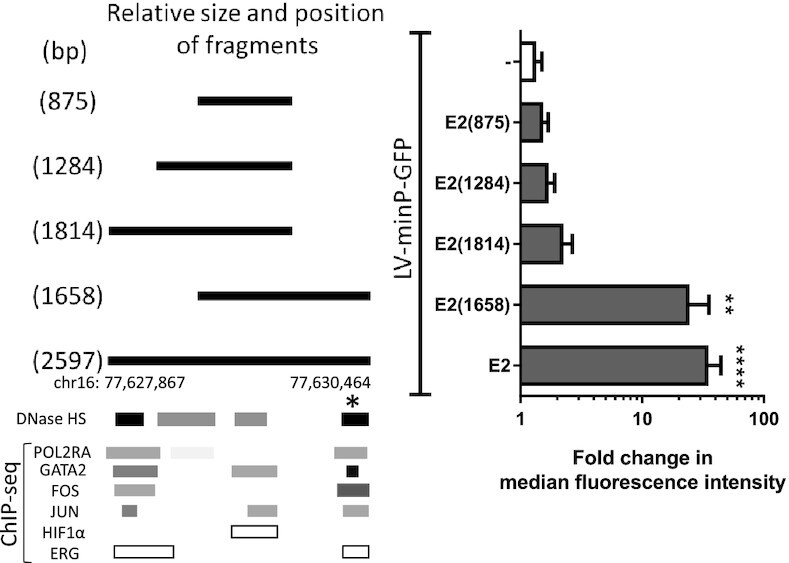
Effects of enhancer size and reporter selection. Vectors were generated containing different genomic lengths (bp size indicated in the name) of the E2 region (shown by solid lines). These fragments corresponded to different clusters of DNaseI hypersensitivity regions and TFs. TFs data was derived from ChIP-seq [TF clusters from ENCODE (83)] for all TFs except HIF1α and ERG, colour intensity is proportional to maximal signal strength. These enhancer fragments were tested in the context of LV-minP-GFP by transduction of EA.hy926. Activity of the regions in regulating GFP expression was determined by flow cytometry. Graphed data is presented as mean ± SEM (*n* = 3), **P < 0.01, ****P < 0.0001.

E2 was unique in its size (2597bp), representing the largest genomic region tested. Furthermore, it encompassed more DNaseI HS sites over other regions like E1. Hence, we tested if by increasing the genomic region of E1, its enhancer activity would become apparent. However, size of the enhancer fragment and inclusion of more DNase I HS sites did not affect E1 activity (Supplementary Figure S2). Vice versa, E2 activity was maintained when its size was decreased to 1.66 kb (Figure 4). However, further reductions of E2 were not tolerated, clearly indicating that E2 activity is determined by sequences within a 1.66 kb region from chr16:77628806–77630464. Interestingly, only one (marked by *) out of the two strong DNaseI HS domains and regions of dense TF binding sites, was required for activity [compare E2(1814) and E2(1658)], and exclusion of that domain as occurs in E2(1814) severely reduced the activity of E2.

### Hypoxia regulates E2 endothelial specific enhancer

**Figure 5.**
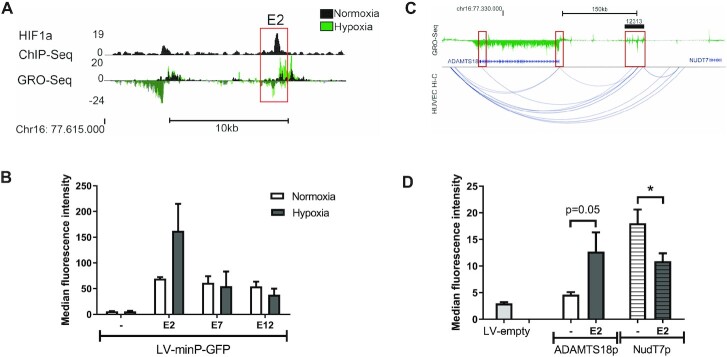
E2 activity is upregulated by hypoxia and it contacts ADAMTS18. (**A**) ChIP-seq analysis of HIF1α binding demonstrates a binding site in E2 and increased eRNA transcription (GRO-seq) following hypoxia treatment. (**B**) HUVECs were transduced with LV-minP-GFP-E2, E7, E12 or control vector and thereafter cells were incubated in hypoxia (1%O_2_, 5% CO_2_) for 24h before GFP quantitation with flow cytometry (mean ± SEM, *n* = 3). (**C)** Hi-C data from HUVECs shows the interaction between SE12313 and *ADAMTS18* gene. GRO-seq track demonstrates nascent RNA expression at SE12313 and *ADAMTS18*. (**D**) EA.hy926 cells were transduced with LV-GFP-E2 or control vectors either encoding the *ADAMTS18* or *NUDT7* promoters and GFP expression determined by flow cytometry. Data is presented mean ± SEM (*n* = 3; ANOVA Tukey's multiple comparison, *P* < 0.05).

For therapeutic endothelial-targeted vectors, it would be beneficial to further induce the transgene expression in hypoxia. HIF-1α is an important transcription factor activated in hypoxia (49). Hence, using HIF-1α CHIP-seq data from HUVECs we assessed the presence of HIF-1α binding site in our selected enhancers and found HIF-1α binding at two of our selected enhancers (Table 1), one of which was in E2 (Figure 5A). Therefore, we investigated the effect of hypoxia on the activity of this enhancer. Following transduction, EC were either incubated in normoxic or hypoxic (1% O_2_) conditions for 24h prior to analysis of GFP expression. Hypoxia induced a further 2-fold activation of E2-induced GFP expression, whereas no change was seen for E7 or E12 in HUVECs (Figure 5B). The hypoxia-induced activation thus correlated with the presence of a HIF-1α binding site in E2 and its absence in E7 and E12.

### The endothelial specific SE contacts ADAMTS18 gene and E2 regulates its promoter

Importantly, we wanted to assess what genes E2 contacts and regulates endogenously. Hi-C analysis of HUVECs (41) demonstrated that SE12313 interacts with the promoter and 3′UTR regions of *ADAMTS18* gene but does not contact the closest gene *NUDT7* (Figure 5C). In addition, SE12313 localized in the same topological associated domain (TAD) with *ADAMTS18*, but not with *NUDT7* (Supplementary Figure S3a). To investigate the functionality of this contact, we tested E2 activity in the context of both neighbouring promoters, *ADAMTS18p* and *NUDT7p*. Interestingly, *ADAMTS18* promoter had low basal activity in EC, but it was activated by E2 (Figure 5D). In contrast, *NUDT7* promoter demonstrated considerable basal activity compared to the other promoters but was not positively regulated by E2, rather its activity was reduced (Figure 5D). The differential regulation of ADAMTS18p by E2 is also consistent with the expression of this gene, which is mainly found in EC and U87 (Supplementary Figure S3b), thus perfectly correlating with the presence of the parent SE SE12313 only in these cells. Overall, these results indicate that E2 enhancer as part of SE12313 regulates *ADAMTS18* gene and validate the functional connection between these two regions seen by the Hi-C data.

### Genomic deletions of endothelial specific enhancers reveal cell cycle and angiogenesis related targets

**Figure 6.**
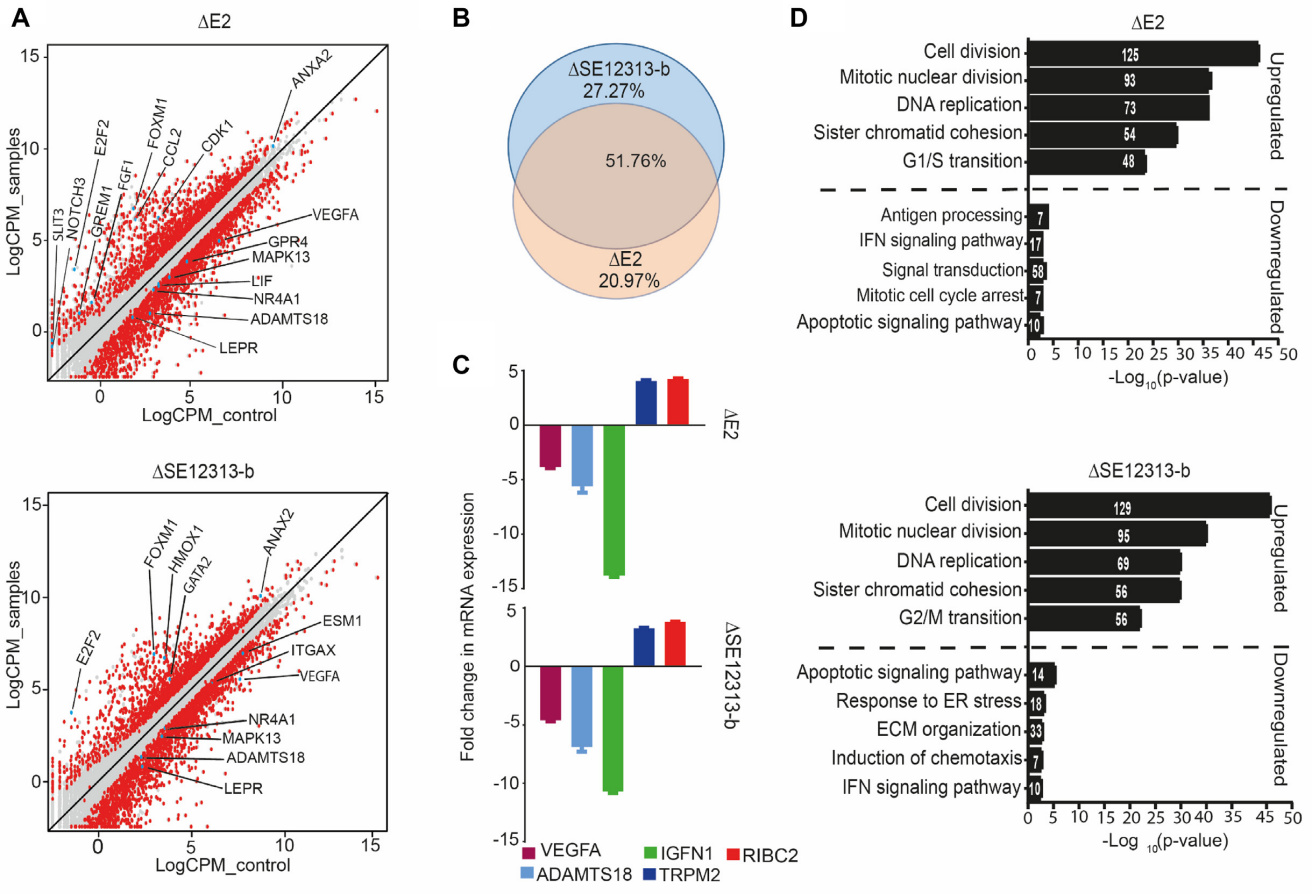
Genome-wide effects of ΔE2 and ΔSE12313-2b. (**A**) Scatter plots representing differentially expressed genes (DEGs). Genes that exhibited ≥2-fold change and 0.05 FDR were considered differentially expressed and are represented by red dots. The genes that have been discussed in the result or discussion sections are highlighted with blue dots. (**B**) Venn diagram demonstrating large overlap between the DEGs from ΔE2 and ΔSE12313-2b. (**C**) RT-qPCR analysis of some of the DEGs identified in the RNA-seq analysis. Data is presented as mean ± SEM of three individual cell lines for each deletion. (**D**) GO ontology analysis identified top biological process linked with upregulated or downregulated genes in ΔE2 (top) and SE12313_bΔ (bottom) cell lines. The number within the bars equates to the number of DEGs detected in the biological process.

To further assess the role of E2 in the context of the cellular genome we deleted E2 and analyzed its effects on transcription genome-wide. In addition, as a negative control an *in vitro* inactive enhancer SE12313-b within SE12313 (see Figure 3A) was also deleted from EA.hy926 genome using CRISPR/Cas9. Following confirmation of the deletion in EA.hy926, first by differential PCR amplicon size and then by Sanger sequencing (Supplementary Figure S4), three clonal knockout lines of each region were subjected to RNA-sequencing. We identified 1984 differentially expressed genes (DEGs; fold > 2 and FDR < 0.05) upon deletion of E2, of which 822 were upregulated and 1162 were downregulated (Figure 6A and supplement Excel file). Similarly, deletion of SE12313-b also resulted in 2156 DEGs, of which 878 were upregulated and 1278 were downregulated. Interestingly, there was close overlap between the DEGs from ΔE2 and ΔSE12313-b with 1412 regulated genes shared in both lists from the total DEGs of 1984 and 2156, respectively (Figure 6B) supporting similarity of action. The 100 most strongly upregulated and downregulated DEGs in ΔE2 and ΔSE12313-b are listed in Tables 2 and 3, respectively. Amongst both upregulated and downregulated genes many important angiogenic molecules were present. For instance, in E2 deletion clones, *NOTCH3*, *CCL2, FGF1, GREM1, ANXA2* and *FOXM1* were upregulated while *VEGFA, MMP10, ITGAX, LEPR* and *NR4A1* were downregulated. Similarly, SE12313-b deletion upregulated genes associated with angiogenesis including *GATA2, ANXA2* and *HMOX1* whereas downregulated genes included *VEGFA, MMP10, LEPR, ESM1* and *NR4A1*. It's of interest that the most upregulated gene in both of the deletions is *TRPM2* which is associated with EC permeability (50). Importantly we also observed downregulation of *ADAMTS18* [–2.8 logFC and –2.4 logFC in ΔE2 and ΔE12313-b, respectively] and no change in *NUDT7*, which is consistent with the evidence presented so far of genomic contact between SE12313 and ADAMTS18 and E2 regulation of its promoter. This reduction in *ADAMTS18* expression as well as the regulation of other genes were independently verified by RT-qPCR (Figure 6C).

**Table 2.** Top 100 differentially expressed genes (DEGs) in ΔE2

Upregulated				Downregulated			
Gene	logFC	Gene	logFC	Gene	logFC	Gene	logFC
*TRPM2*	8.2	*MYO5C*	5.1	*IGFN1*	–10.2	*SBK2*	–5.3
*RIBC2*	7.9	*BIRC5*	5.1	*NCAM2*	–9.5	*CD74*	–5.2
*OPCML*	7.4	*ANLN*	5	*VIPR1*	–8.2	*ST3GAL6*	–5.2
*FAM111B*	7.2	*DLGAP5*	5	*KLHDC7B*	–8.0	*SBK3*	–5.1
*DRGX*	7.1	*AURKB*	4.9	*MRC2*	–7.9	*EEF1A2*	–5.1
*ESCO2*	7.0	*LOC105373354*	4.9	*MYOM1*	–7.8	*MDGA2*	–5.1
*HIST1H1T*	6.7	*ZWINT*	4.9	*TMEM132D-AS1*	–7.7	*PSD*	–5.1
*VIT*	6.5	*ASPM*	4.9	*SOX8*	–7.5	*KIF21B*	–5.1
*RRM2*	6.5	*JPH3*	4.9	*SCN3A*	–7.4	*MUC15*	–5.0
*LINC00565*	6.5	*CCR4*	4.8	*SLC2A14*	–7.3	*TM4SF19*	–5.0
*SPC25*	6.3	*CDCA8*	4.8	*ATG9B*	–7.3	*LIPC*	–5.0
*E2F2*	6.3	*GTSE1*	4.8	*MMP10*	–7.3	*LYRM4-AS1*	–5.0
*ORC1*	6.2	*CYB5R2*	4.8	*PIK3C2G*	–7.3	*ABAT*	–5.0
*TK1*	6.2	*TTK*	4.7	*CFH*	–7.0	*ACP7*	–5.0
*NOTCH3*	6.1	*TMSB15A*	4.7	*PPL*	–7.0	*SHANK2*	–5.0
*SLITRK5*	6.1	*GINS4*	4.7	*C6orf223*	–7.0	*UCN*	–4.9
*MYBL2*	6.0	*KIFC1*	4.6	*MYO15A*	–6.9	*ZPLD1*	–4.9
*ERCC6L*	6.0	*E2F8*	4.6	*LINC01611*	–6.7	*CLSTN2*	–4.9
*ITGB2-AS1*	6.0	*NDC80*	4.6	*PDE7B*	–6.7	*CYGB*	–4.9
*WDFY4*	6.0	*KIF15*	4.6	*FER1L6*	–6.7	*CHPF*	–4.8
*FREM3*	5.9	*AQP3*	4.6	*PLCZ1*	–6.6	*LOC100506098*	–4.8
*SORL1*	5.9	*FOXM1*	4.5	*LINC02141*	–6.6	*LRRC75B*	–4.8
*SHCBP1*	5.7	*DDIAS*	4.5	*TMEM178A*	–6.6	*PRSS27*	–4.7
*ZNF114*	5.6	*CEP55*	4.5	*B3GNT7*	–6.5	*CHRNE*	–4.7
*LOC643201*	5.6	*HIST1H2BL*	4.5	*HHIPL1*	–6.5	*KCP*	–4.7
*LINC01291*	5.5	*TOP2A*	4.5	*MEDAG*	–6.3	*NHEG1*	–4.6
*NCAPG*	5.5	*FBXO43*	4.5	*HLA-DRB1*	–6.2	*NLRC4*	–4.6
*KIF18B*	5.4	*PRSS35*	4.4	*MATK*	–6.2	*SPATA17*	–4.6
*SKA1*	5.4	*CDCA5*	4.4	*LOC105369165*	–6.1	*FSIP2*	–4.6
*PBK*	5.4	*MT1E*	4.4	*SLC22A31*	–6.1	*SERPINB7*	–4.6
*MCM10*	5.3	*ZNF367*	4.4	*MAGEB18*	–6.1	*CLIC2*	–4.6
*MKI67*	5.3	*DTL*	4.4	*INHBE*	–6.1	*OSCAR*	–4.5
*TCF19*	5.3	*CSTF3-DT*	4.4	*ATP6V0D2*	–6.0	*LINC02609*	–4.5
*HIST1H3J*	5.3	*KSR2*	4.4	*PRDM9*	–6.0	*B3GNT5*	–4.5
*OIP5*	5.3	*CARD11*	4.4	*LONRF3*	–6.0	*MID2*	–4.4
*METTL7A*	5.3	*MYB*	4.4	*FSCN2*	–5.8	*LMTK3*	–4.4
*HIST1H1B*	5.3	*DEPDC1*	4.4	*STC1*	–5.8	*LST1*	–4.3
*PADI2*	5.3	*NUF2*	4.4	*IL1B*	–5.7	*SOCS2-AS1*	–4.3
*PIMREG*	5.2	*LBH*	4.3	*TNC*	–5.7	*MIR616*	–4.3
*APOBEC3B*	5.2	*BUB1*	4.3	*LOC286178*	–5.7	*SLC16A7*	–4.3
*KIF20A*	5.2	*ASB9*	4.3	*LOC100505664*	–5.6	*CACNA2D4*	–4.2
*SLIT3*	5.2	*SGO1*	4.3	*PPP1R32*	–5.4	*FAM131C*	–4.2
*PCLAF*	5.2	*KIF2C*	4.3	*ASTN1*	–5.4	*ADAM21*	–4.2
*EXO1*	5.2	*CCNB2*	4.3	*BAIAP3*	–5.4	*ALDH1A1*	–4.2
*HIST1H2BJ*	5.2	*POLQ*	4.3	*XAF1*	–5.4	*MAP2*	–4.2
*FAM222A*	5.2	*CDCA3*	4.3	*COLEC12*	–5.3	*LOC101927040*	–4.1
*GINS2*	5.2	*MPP2*	4.3	*NRXN1*	–5.3	*DMKN*	–4.1
*CENPA*	5.1	*XRCC2*	4.3	*GTSF1*	–5.3	*LINC00648*	–4.0
*NCAPH*	5.1	*CDC6*	4.3	*NFE4*	–5.3	*LINC02352*	–4.0
*CDC20*	5.1	*MYBL1*	4.3	*PMEPA1*	–5.3	*CFL1P1*	–4.0

**Table 3.** Top 100 differentially expressed genes (DEGs) in ΔSE12313-b

Upregulated				Downregulated			
Gene	logFC	Gene	logFC	Gene	logFC	Gene	logFC
*RIBC2*	7.6	*HIST1H2BJ*	4.9	*EPB41L3*	–12.3	*PRLR*	–5.7
*TRPM2*	7.6	*CEP55*	4.8	*PXDN*	–11.5	*RIT2*	–5.7
*SLITRK5*	7.2	*GTSE1*	4.8	*BEST1*	–8.7	*LINC01477*	–5.7
*ESCO2*	6.9	*FCER2*	4.8	*CHRNE*	–8.7	*DAPK1*	–5.7
*ZNF114*	6.8	*AURKB*	4.7	*THSD7B*	–8.6	*CFB*	–5.7
*LINC01291*	6.7	*KIF15*	4.7	*CNTNAP4*	–8.5	*IL1B*	–5.7
*VASN*	6.5	*CORO1A*	4.7	*TMEM178A*	–8.4	*LCP2*	–5.6
*DRGX*	6.4	*CDC20*	4.7	*VIPR1*	–8.2	*HSD11B1*	–5.6
*E2F2*	6.2	*KIFC1*	4.7	*NLRC4*	–8.1	*SLC6A1*	–5.6
*C10orf90*	6.2	*GINS2*	4.6	*MAGEC2*	–7.7	*SYT12*	–5.6
*RRM2*	6.2	*ASPM*	4.6	*CYBA*	–7.7	*PLCB2*	–5.6
*ORC1*	6.2	*PKMYT1*	4.6	*LINC01425*	–7.7	*WAS*	–5.5
*FAM111B*	6.2	*PLPP4*	4.6	*LOC100505664*	–7.5	*UPB1*	–5.5
*TK1*	6.1	*ANLN*	4.5	*LOC105376105*	–7.4	*NHEG1*	–5.4
*RAET1G*	6.1	*CDCA3*	4.5	*THY1*	–7.4	*MUC15*	–5.4
*METTL7A*	6.1	*RASL11A*	4.5	*ATG9B*	–7.3	*MATK*	–5.4
*SORL1*	6.0	*CDCA8*	4.5	*LUZP2*	–7.3	*GPR158*	–5.4
*MYBL2*	6.0	*GINS4*	4.4	*PPP1R32*	–7.3	*BMPER*	–5.3
*SPC25*	6.0	*HIST1H3J*	4.4	*FAM9B*	–7.2	*PTPRO*	–5.3
*LINP1*	6.0	*ASB9*	4.4	*SYT9*	–7.2	*SELENOP*	–5.3
*AQP3*	5.9	*CCNB2*	4.4	*IGFN1*	–7.2	*MAMDC2*	–5.3
*VIT*	5.9	*NDC80*	4.3	*MAP6*	–7.1	*NFE4*	–5.3
*LINC00565*	5.7	*DTL*	4.3	*IGSF9B*	–7.1	*LINC01344*	–5.3
*ITGB2-AS1*	5.7	*SGO1*	4.3	*ARLNC1*	–7.0	*MRC2*	–5.3
*MCM10*	5.7	*HIST1H2BL*	4.3	*SRGAP3*	–7.0	*CTSO*	–5.3
*SERPINF2*	5.7	*NUF2*	4.3	*LINC01060*	–7.0	*FER1L6*	–5.2
*PCLAF*	5.6	*CDC6*	4.3	*SOCS2-AS1*	–6.9	*MYOM1*	–5.2
*MKI67*	5.6	*TOP2A*	4.3	*INHBE*	–6.8	*SPEF2*	–5.2
*ERCC6L*	5.5	*HIST1H3D*	4.2	*ACP7*	–6.8	*ND1*	–5.1
*KIF18B*	5.3	*LRRC7*	4.2	*PDE7B*	–6.7	*LUCAT1*	–5.1
*SHCBP1*	5.3	*KIF11*	4.2	*XIST*	–6.7	*ITGA2B*	–5.0
*APOBEC3B*	5.3	*KIF2C*	4.2	*IGFBP3*	–6.7	*LYRM4-AS1*	–5.0
*HIST1H1B*	5.3	*E2F8*	4.2	*DCT*	–6.6	*GALNT3*	–4.9
*TCF19*	5.2	*FAM83D*	4.2	*MACROD2*	–6.6	*LINC00313*	–4.9
*OIP5*	5.2	*LOC105373354*	4.2	*LINC02141*	–6.6	*SOX8*	–4.9
*EEPD1*	5.2	*HMMR*	4.2	*FSIP2*	–6.4	*MMP10*	–4.9
*EXO1*	5.2	*CDK1*	4.2	*ARHGAP9*	–6.4	*SGCZ*	–4.9
*KSR2*	5.1	*BUB1*	4.1	*CEACAM22P*	–6.3	*ADAMTS20*	–4.9
*PBK*	5.1	*CDCA2*	4.1	*KLHDC7B*	–6.2	*ZMAT1*	–4.8
*SKA1*	5.1	*DLEU2*	4.1	*LINC01619*	–6.2	*SLC17A7*	–4.8
*ZWINT*	5.1	*CDCA5*	4.1	*XAF1*	–6.2	*SCN3A*	–4.8
*PIMREG*	5.1	*SPRR2B*	4.1	*TNC*	–6.1	*LGR6*	–4.8
*KIF20A*	5.1	*CENPF*	4.1	*SLC22A31*	–6.1	*GOLGA8R*	–4.7
*BIRC5*	5.1	*POLQ*	4.1	*MAGEB18*	–6.1	*MUC16*	–4.7
*FAM222A*	5.0	*NEK2*	4.1	*LINC02167*	–6.1	*STC1*	–4.7
*TMSB15A*	5.0	*HIST1H2A*	4.1	*HOXB9*	–6.0	*HHIPL1*	–4.7
*CENPA*	5.0	MPP2	4.0	*MAP2*	–6.0	LIPC	–4.7
*NCAPG*	5.0	AKAP5	4.0	*STXBP5-AS1*	–5.9	BAIAP3	–4.6
*DLGAP5*	5.0	PRC1	4.0	GRIN3B	–5.8	TGFBR3L	–4.6
*DACT3*	5.0	SKA3	4.0	SHANK2	–5.8	CCDC146	–4.6

By further assessment of other potential *cis*-targets located within ≤ 1Mb of the deleted region, we identified three genes including *CNTNAP4* (–8.4 logFC), *WWOX* (–1.3 logFC) and *SYCE1L* (–1.3 logFC) that were downregulated in Δ*SE12313*-b while *VAT1L* (–2.6 logFC) was downregulated in ΔE2. Of these only SYCE1L was located in the same topological domain as SE12313 (Supplementary Figure S3a). To identify relevant biological processes linked with DEGs we performed gene ontology (GO) analysis with upregulated and downregulated genes, independently. The topmost biological processes linked with upregulated genes where shared in E2 and SE12313-b deletions; and these included cell division, mitotic nuclear division, DNA replication, sister chromatid cohesion and cell-cycle phase transition (Figure 6D). Similarly, two biological processes linked with downregulated genes including IFN-mediated signalling pathway and apoptotic signalling were also shared in E2 and SE12313-b deletions.

To further explore the mechanisms that may explain the observed differential expression, we used the upstream regulator analysis from Ingenuity Pathway Analysis (IPA; Qiagen). The list of top upstream regulators associated with DEGs in ΔSE2 and ΔSE12313-b are shown in Tables 4 and 5, respectively. The majority (approximately 70%) of identified upstream regulators as well as their activation state were shared by both enhancer deletions. Among them included cell cycle regulators such as TP53, E2F1, E2F2, E2F3, CDKN1A and RBL1 as well as factors involved in angiogenic pathway such as HGF, VEGF and FOXM1. Altogether, our genome-wide analysis of ΔE2 and ΔSE12313-b revealed several downstream targets implicated in important endothelial related functions including cell cycle and angiogenesis. In contrast to the differential reporter assay results, both individual enhancers of SE12313 demonstrated highly similar effects on genome-wide transcriptomes of ECs.

**Table 4.** Top 20 upstream regulators upon deletion of E2

Regulator	Molecule type	Activation state	*z*-score	*P*-value
HGF	growth factor	Activated	2.5	3.35E–26
E2F3	transcription regulator	Activated	4.6	5.98E–19
FOXM1	transcription regulator	Activated	5.6	1.22E–17
VEGF	growth factor	Activated	3.0	1.15E–15
E2F1	transcription regulator	Activated	5.2	3.04E–14
AREG	growth factor	Activated	3.1	1.90E–13
CDKN1A	kinase	Inhibited	–4.0	4.01E–13
KDM5B	transcription regulator	Inhibited	–4.3	1.54E–12
E2F2	transcription regulator	Activated	2.5	3.22E–12
RBL1	transcription regulator	Inhibited	–4.3	2.11E-11
TP53	transcription regulator	Inhibited	–5.1	2.24E–11
TP73	transcription regulator	Inhibited	–4.1	2.05E–10
SMARCB1	transcription regulator	Inhibited	–4.7	6.80E–09
RB1	transcription regulator	Inhibited	–4.2	7.66E–09
E2F	transcription regulator	Activated	3.3	5.88E-08
SP600125	chemical kinase inhibitor	Activated	2.1	9.64E–07
EIF4G1	translation regulator	Activated	2.7	1.41E–06
Rb	tumor suppressor	Inhibited	–3.0	3.65E–06
DDIT3	transcription regulator	Activated	3.0	1.37E–05
SMOC2	growth factor	Activated	2.4	1.44E–05

**Table 5.** Top 20 upstream regulators upon deletion of SE12313-b

Regulator	Molecule type	Activation state	*z*-score	*P*-value
HGF	growth factor	Activated	3.0	8.09E–26
VEGF	growth factor	Activated	3.5	3.95E–21
FOXM1	transcription regulator	Activated	5.1	1.01E–16
E2F1	transcription regulator	Activated	4.5	5.37E–15
TP53	transcription regulator	Inhibited	–5.4	2.40E–14
E2F3	transcription regulator	Activated	4.1	4.60E–14
E2F2	transcription regulator	Activated	2.3	1.10E–12
KDM5B	transcription regulator	Inhibited	–4.3	2.77E–12
RBL1	transcription regulator	Inhibited	–4.0	8.24E–11
AREG	growth factor	Activated	3.4	2.28E–10
TP73	transcription regulator	Inhibited	–3.6	7.26E–10
F2	peptidase	Inhibited	–2.1	1.10E–09
RB1	transcription regulator	Inhibited	–4.1	6.36E–09
E2F	transcription regulator	Activated	3.2	2.15E–08
SMARCB1	transcription regulator	Inhibited	–4.6	3.39E–08
CDKN1A	kinase	Inhibited	–4.1	6.93E–08
TGFB1	growth factor	Inhibited	–3.0	1.63E–07
SP600125	chemical kinase inhibitor	Activated	2.9	1.75E–06
TNF	cytokine	Inhibited	–3.5	3.56E–06
DDIT3	transcription regulator	Activated	2.5	3.66E–06

### Deletion of E2 and SE12313-b reduce cell proliferation

**Figure 7.**
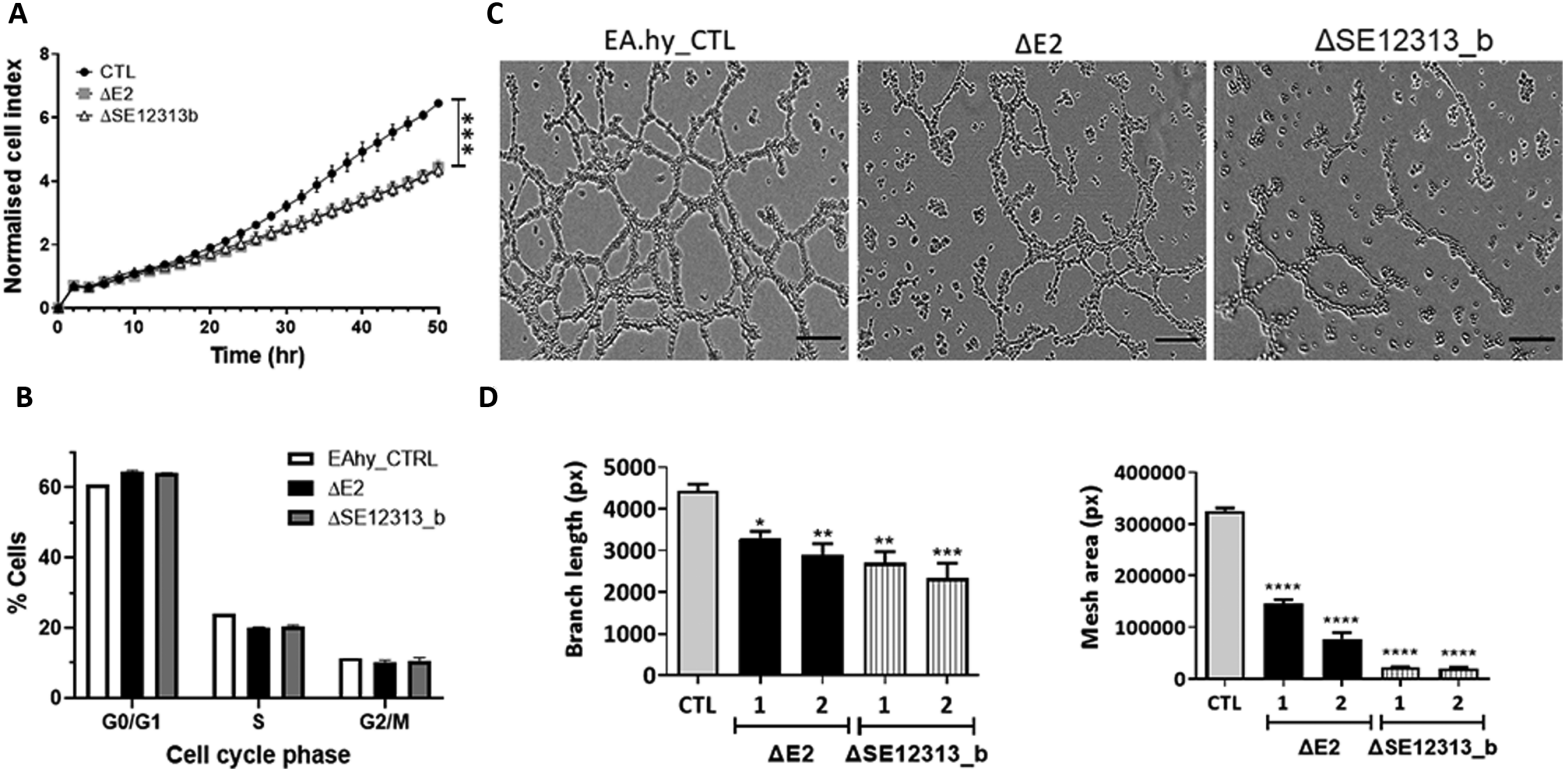
Effect of E2 and SE12313-b deletions on cell proliferation and tube formation. (**A**) Cell proliferation assay using the XCelligence DP system over 52h for ΔE2 and ΔSE12313-b clones (3 for each deletion) and EA.hy926 CTL cell line. Data presented as mean ± SEM (*n* = 3; multiple ANOVA *** *P* < 0.0001). (**B**) Cell cycle analysis of ΔE2 and ΔSE12313-b clones (three for each deletion) and EA.hy926 CTL cell line. The data represents the average percentage of cells in the different phases of the cell cycle. Data is presented as mean ± SEM (*n* = 4). (**C**) Images of tube/mesh formation (16 h) for ΔE2 and ΔSE12313-b clones and EA.hy926 CTL cell line. The scale bars correspond to 100 μm. (**D**) Quantitative analysis (Image J) of endothelial cell branches and area in two clones (1, 2) of each deletion (ΔE2 and ΔSE12313-b) and in EA.hy926 CTL cell line. Data presented as mean ± SEM (*n* = 9 images from three independent experiments); one-way ANOVA * *P* < 0.05, ** *P* < 0.005, *** *P* < 0.0005, **** *P* < 0.0001).

To further assess the effects of E2 and SE12313-b deletion on cell proliferation and to confirm the results derived from the RNA-sequencing, we performed cell proliferation assay on these cell lines (months after the RNP transfection to avoid any proliferation affect due to the CRISPR/Cas9 double-strand break). The results demonstrated that the deletion of E2 or SE12313-b induced a significant (*P* < 0.0001) reduction in cell proliferation compared to the control cells, which had been transfected with gRNAs and cultured in parallel to the clones during their derivation (Figure 7A). Interestingly, the reduction in cell proliferation was similar in both ΔE2 and ΔSE12313-b cell lines, consistent with the strong overlap observed in differentially regulated genes affecting cell division. To further investigate the mechanism of the observed reduction of cell proliferation, we analysed the cell cycle stages in ΔE2 and ΔSE12313-b lines. As presented in Figure 7B, cell cycle analysis of ΔE2 and ΔSE12313-b clones showed an increase in the percentage of cells in G0/G1 and a corresponding decrease in the number of cells in S-phase, as compared to control cells. These results are consistent with the decreased proliferation of the cells and the predicted cell-cycle processes affected by the differentially upregulated genes in these clones. In addition, in ΔE2 clones there was a small but coherent decrease in the number of cells in G2/M phase, these changes in cell cycle phases were observed in all clones that were tested (Supplementary Table S5).

Further assessment of DEGs in ΔE2 and ΔSE12313-b cell lines using Gene set Enrichment analysis (GSEA) from Molecular Signatures Database (MSigDB), we found a set of downregulated genes including *VEGFA, CYBA, NR4A1, SEMA5A, SCG2, ITGA4, DYSF and VEGFC* that are linked with positive endothelial cell proliferation (GO:0001935). In addition to this analysis, a set of upregulated genes including *AURKA, BLM, CCNF, CDT1,CHEK1, WEE1, MAD2L1, RAD21, RRM2, E2F2, E2F7 and E2F8* emerged with the genes that are known to participate in negative regulation of cell cycle (GO:0045786; Supplementary Table S6). Together, these data suggest that E2 and SE12313-b play a role in cell proliferation and cell cycle progression by regulating directly or indirectly some of the genes that are associated with these biological processes.

### Deletion of E2 and SE12313-b affects tube formation

SE12313 effects on cell proliferation and regulation of genes such as *VEGFA* may in turn affect the endothelial angiogenic function. To investigate this further, we tested the enhancer deleted clones in a tube formation assay. As can be clearly seen in Figure 7B, the enhancer deletions resulted in a dramatic change in the tube formation ability by these cells. There was a significant reduction in both the tube lengths and mesh area (Figure 7C).

**Figure 8.**
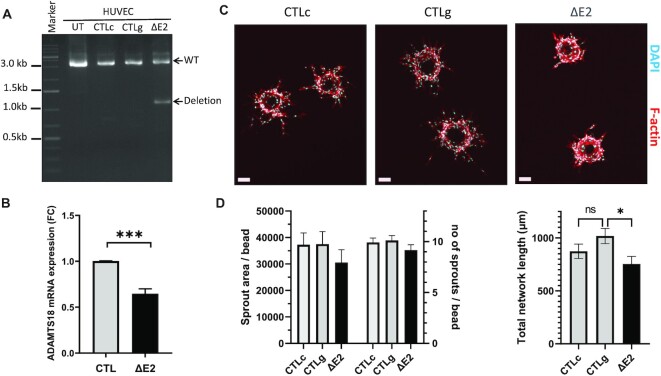
Effect of ΔE2 on ADAMTS18 expression and angiogenesis in HUVEC. (**A**) PCR analysis of genomic DNA extracted from ΔE2 cells and in control cells including untreated (UT), CRISPR/Cas9 RNP transfected cells in combination with one gRNA (CTLc) only and in cells transfected with only two gRNAs without CRISPR/Cas9 (CTLg). The deletion band corresponds to 1115bp while WT band corresponds to 3606 bp. (**B**) Expression analysis of ADAMTS18 expression upon ΔE2. Data is presented as mean ± SEM (*n* = 3; unpaired *t*-test *** *P* < 0.001). (**C**) Representative images of angiogenesis bead assay for ΔE2 and HUVECs control cells (day3) stained with phalloidin-A635 (F-actin, red) and DAPI (blue). (**D**) Quantitative analysis (ImageJ) of endothelial sprouts of ΔE2 (*n* = 23 beads) and HUVEC controls CTLc (*n* = 25 beads) and CTLg (*n* = 26 beads). Data is presented as mean ± SEM (One way ANOVA and Tukey's multiple comparisons test * *P* < 0.05).

To confirm these findings, E2 deletion was performed in primary endothelial cells, in HUVEC. Using the same CRISPR/Cas9 RNP transfection we were able to achieve a 40% deletion efficiency (Figure 8A). In these cells we observed reduced ADAMTS18 mRNA expression (Figure 8B), albeit to a lower degree than in the EAhy.926 clones which is likely due to the mixed population of cells. Likewise, in a 3D angiogenic assay we also saw an effect on sprout formation (Figure 8C), which manifested in a trend towards reduced sprout number and area as well as a significant reduction in total network length (Figure 8D).

### SE12313 downregulates ADAMTS18 which affects endothelial tube formation

**Figure 9.**
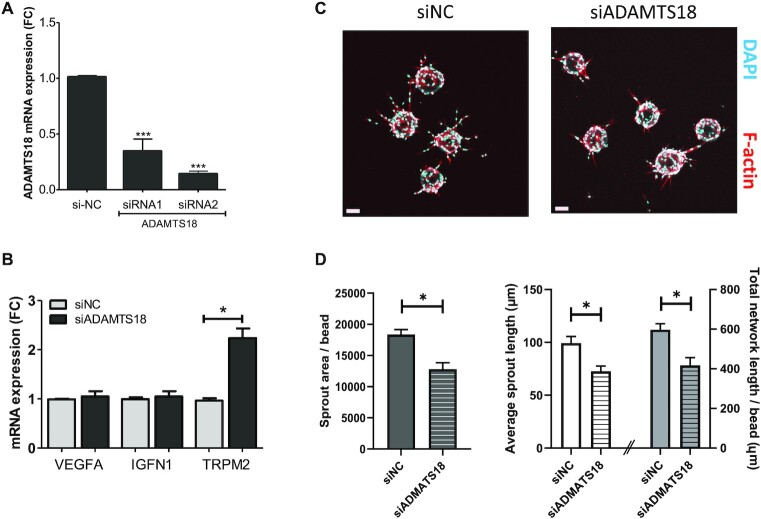
Knockdown of ADAMTS18 mRNA and its effect on angiogenesis. (**A**) EA.hy926 cells transfected with DsiRNA specific to ADAMTS18 mRNA (siRNA 1 and 2) resulted in reduced ADAMTS18 mRNA as compared to control DsiRNA (siNC) transfected cells (RT-qPCR; One way ANOVA, *** *P* < 0,0001). (**B**) Gene expression in cells with DsiRNA (siRNA2)-reduced ADMATS18 mRNA (siADAMTS18) compared to siRNA-NC transfected cells (siNC). Gene expression was normalized to ACTB expression. QPCR data is presented as mean ± SEM (*n* = 3; two-way ANOVA, * *P* < 0.05). (**C**) Representative images of angiogenesis bead assay for siNC and siADAMTS18-transfected HUVECs (day3) stained with phalloidin-A635 (F-actin, red) and DAPI (blue). (**D**) Quantitative analysis (ImageJ) of endothelial sprouts protruding from beads of siNC (*n* = 21 beads) and siADAMTS18 (*n* = 23 beads) treated ECs. Data is presented as mean ± SEM and was analysed by unpaired *t*-test *P < 0.05.

To determine if some of the gene expression changes seen following enhancer deletions are due to SE12313 cis-regulation of *ADAMTS18*, we performed knockdown of *ADAMTS18* mRNA using dicer-substrate (D)siRNAs. In EAhy.926, we achieved >80% knockdown of *ADAMTS18* mRNA (Figure 9A), and this resulted in an increase in *TRPM2* mRNA as was seen with SE12313 enhancer deletions (Figure 9B). However, other genes products including *VEGFA* and *IGFN1* were unaffected, supporting the view that only some of SE12313 effects are via its regulation of *ADAMTS18*. In addition, downregulation of *ADAMTS18* did not significantly affect cell proliferation (Supplementary Figure S5a) indicating that SE12313 effects on cell proliferation are independent of its *ADAMTS18* regulation.

*ADAMTS18* is a secreted metalloprotease that has been recently localized in epithelial branch-tip cells and implicated in branching morphogenesis of epithelial cells (51) as well as angiogenesis (52). Hence, SE12313 regulation of *ADAMTS18* may affect endothelial tube formation. To test this, HUVECs with DsiRNA-mediated reduction in *ADAMTS18* expression (Supplementary Figure S5b) were used in an angiogenesis bead assay. We clearly observed an effect of *ADAMTS18* mRNA repression on EC sprouting (Figure 9C). Although the sprout numbers were similar between Dsi-NC and Dsi-ADAMTS18 treated cells (6.19 ± 0.28, *n* = 21 and 5.78 ± 0.36, *n* = 23, respectively), there was a significant reduction in the sprout area, average sprout length and the overall network length per bead (Figure 9D). These data unequivocally demonstrate a functional effect of *ADAMTS18* on endothelial sprouting and reveal that SE12313 regulation of this important endothelial function is due to its regulation of *ADAMTS18*.

## DISCUSSION

In this study we have functionally identified and characterized an endothelial-specific enhancer derived from SE12313. This enhancer regulated the expression of reporter genes encoded in lentiviral vectors, specifically in ECs. Using CRISPR/Cas9 deletion and genome-wide RNA sequencing, we identified hundreds of genes regulated by this enhancer and found that they impact cell cycle, proliferation and angiogenesis. Importantly we measured reduced proliferation and modulation of tube formation and endothelial sprouts following the enhancer deletions both in EA.hy926 and in primary HUVEC, respectively. Significantly, we also identified *ADAMTS18* as the cis-gene target of SE12313 and demonstrated that *ADAMTS18* can affect endothelial sprouting, consistent with an important role for this SE and *ADAMTS18* in endothelial cell biology.

The readout of SE12313 specificity may be defined by the genes that it regulates. Using Hi-C, we detected chromatin interaction between SE12313 and *ADAMTS18* gene. We confirmed the regulation of *ADAMTS18* by E2/SE12313, both by demonstrating *in vitro* activation of *ADAMTS18* promoter by E2 in reporter assay and reduction in *ADAMTS18* gene expression following deletion of E2 and SE12313_b enhancers. Consistently, *ADAMTS18* RNA expression is enriched in ECs and U87 cells (53), concomitant with SE13231 presence in these same cell lines. Importantly, we identified a role for *ADAMTS18* in endothelial sprouting. Although ADAMTS18 was initially associated with cancer (54) and thrombosis (55), recently ADMATS18 deficiency in zebrafish (52) or in mice (56) were associated with observations of defective angiogenesis and vascular malformations, respectively. In addition, recent studies have implicated *ADAMTS18* in epithelial tube formation. *ADAMTS18* mRNA was localized to epithelial branch-tip progenitor cells and was demonstrated to play a role in branching morphogenesis of kidney and lung (51). Likewise, in mammary epithelium Adamts18 is required for stem cell activation and its knockout was associated with reduced or delayed epithelial branching which correlated with impaired Hippo Signalling (57). It is of interest that in E2 and SE12313-b enhancer deletions in ECs at least 5 genes (STK4, NF2, TAZ, TEAD3 and CTGF) involved in Hippo signalling were differentially regulated. Activation of Hippo signalling in EC is known to promote vascular growth and remodelling (58). Other angiogenic signalling pathways are also likely to be involved as expression of *NOTCH3, MAPK13, SLIT3* and *LIF* were altered following deletion of the SE12313 enhancers. Likewise, the well-known angiogenic factor *VEGF* was also decreased in both enhancer deletions. Previously, we have shown that overexpression of the short form of VEGFD in EC significantly increased *ADAMTS18* (59), demonstrating the interplay between *VEGFs* and *ADAMTS18*. Overall, these findings support an important role for *ADAMTS18* and SE12313 in ECs.

From the 20 enhancers that were tested only E2 was specific to EC and activated the expression of reporter genes. This begs the question, what distinguishes E2 from the other enhancers screened? In fact, E2 did not stand out in many parameters, such as abundance of eRNA transcription, level of enrichment of H3K27ac deposition, number of DNaseI hypersensitivity sites or repertoire of TF binding. This profile of E2 is reminiscent of hub enhancers, which likewise were found to be associated with high activity but moderate if any differences in active enhancer modifications or presence of lineage TF as compared to other non-hub enhancers (60). The specificity of E2 is likely to be determined by the TFs it binds, these include JUN, cFOS, cMyc, GATA2 and ERG which are TFs enriched in endothelial-specific SE (21,48) and which have been associated with endothelial cell gene regulation (61,62). Although it is likely that the cooperation of these TF determines the activation of SE12313 and contribute to its specificity, ERG is of particular interest. ERG was previously associated with EC migration and angiogenesis (63) and in the regulation of the key endothelial Notch signalling (62). In addition, Kalna *et al.* (48) were able to identify endothelial SE using ERG enrichment as the ranking parameter and in this list of 1125 endothelial SE, SE12313 ranked at 286 based on its’ ERG occupancy. Furthermore, siRNA knockout of ERG resulted in downregulation of ADAMTS18 (64), which further supports its role in SE12313 activation.

In this study, we also functionally validated the individual enhancers within clusters of SE. In both of the SE that we analysed we observed a clear dominance of activity by one enhancer within a cluster in the reporter assay, as has previously been observed for some SE (65,66), but not others (67,68). Previously a hierarchical organization of SE was proposed (69) and ‘hub’ enhancers within a SE were associated with higher activity (60). This hierarchical organization of SE may explain why the majority of enhancers that we analysed failed to demonstrate activity in our reporter assay. However, super-enhancers hierarchical organization does not exclude contribution of some or all enhancers within a SE, and as such it was demonstrated that combinatorial deletion of enhancers within a SE led to a greater effect on gene expression (66,69). This collaborative activity of enhancers within a SE is also reflected in our data from the enhancer deletions, whereby deletion of either enhancer resulted in similar gene regulation of approximately 2000 genes, in the range reported in previous enhancer deletion studies (70,71). Thus, the close overlap of differentially regulated genes would indicate that most of the enhancer activity is related to the SE activity as a whole, and perturbation of any enhancer within the cluster may negatively affect the SE activity.

Since the tested enhancers were selected according to the highest eRNA expression within the cluster, our results then would indicate that eRNA expression does not correlate with enhancer activity in vectors. This is exemplified by E1 and E2, E1 was associated with the highest level of eRNA in SE12313 and yet had no activity in the reporter assay in contrast to E2 from the same SE, which had high activity but lower transcribed eRNA. Likewise, we did not observe any correlation with the strength of H3K27ac deposition and enhancer activity, as has previously been observed for constitutive enhancers (70,72). Previously, in a systematic comparison of *in vitro* enhancer assays and ENCODE annotations for 2236 enhancers only low correlations were detected (73). In addition, often in genome wide super-enhancer/enhancer studies, these elements are associated with the genes closest to them, however as we have demonstrated in this study, SE13213 did not regulate its closest proximity gene, *NUDT7* but regulated *ADAMTS18*, this then also brings into question the accuracy of this practice in genome wide enhancer-gene association studies. SE13213 and *ADATMTS18* were in the same topological domain and this might be a better way to associate enhancers and genes. In combination, these data highlight the need for functional validation of enhancers.

It is of interest that an enhancer region that was inactive in the *in vitro* screen, SE12313_b, when deleted by CRISPR/Cas9 demonstrated an equal effect on gene regulation as the active E2. This begs the question, why the discrepancy in enhancer activities in different assays? Of course synergistic effects of enhancer constituents within a SE may explain the difference, such that when analysed in isolation, SE12313_b may not be sufficient to increase gene expression, but when removed from the cluster of enhancers and the subsequent removal of factors that it recruits can reduce the activity of the SE as a whole (74). However, it's likely to be multifactorial and can include limitations in either the *in vitro* assay or the CRISPR/Cas9 deletions. One possible limitation of our *in vitro* assay is the integration site of the lentiviral vectors which could affect the enhancer activity. Likewise, the CRISPR/Cas9 deletions may have resulted in greater disruption of the chromatin architecture, thereby affecting enhancer activity. We have recently observed a similar discrepancy between *in vitro* reporter assays and genomic deletion of enhancers. However, in this case enhancers showed activity *in vitro* but not following CRISPR/Cas9 deletion (71). What is clear is that *in vitro* assays alone are insufficient to analyse enhancer activity, and multiple approaches are required to adequately investigate these regulatory regions.

Super-enhancers are dynamic and are remodelled in response to change (69,72,75–78). Likewise, SE12313 was altered by hypoxia. We observed that E2 contained a HIF-1α binding site and in hypoxia, transcription at SE12313 was increased. Furthermore, in the context of the LV vector E2 increased reporter expression following hypoxia. We have recently demonstrated that hypoxia significantly regulates SE genome-wide in ECs, in particular those found near angiogenesis related genes (79). Hypoxia regulation is also very useful in therapeutic vectors. Hypoxia is a common feature of tumours so hypoxia responsive vectors would be beneficial for cancer-specific transgene delivery targeting the tumour endothelium (80). Likewise, hypoxia-regulated endothelial-specific vectors would be useful for angiogenic therapies (6). These therapies are needed for the treatment of infarctions which are caused by blood vessel blockages leading to disruption of blood flow, tissue hypoxia and eventually cellular death. Our endothelial-specific, hypoxia-regulated vector could thus prove very useful for angiogenic-based therapies, as it would only express the therapeutic transgene in the ECs, and it would be further induced by hypoxia.

The endothelial specificity of our vectors could prove valuable as previous development of vectors containing endothelium-specific promoters have met with limited success and generally tissue-specific promoters have demonstrated leakiness and have been associated with low expression (9,81). The endothelial specificity of the vectors should limit non-specific transgene expression in other cell-types and thereby increase safety and limit genotoxicity. In addition, in our current vector only a minimal TATA box sequence is included for the promoter with non or minimal activity observed, which improves vector safety by exclusion of a strong promoter. In the past it has been shown that promoter-less vectors have a reduced chance of neighbouring oncogene activation and hence better safety profiles (82).

Overall, in this study we have provided a detailed characterization of a functionally important SE and demonstrate the potential of our approach in increasing the understanding of the role of gene regulatory elements important for EC biology. To this end, we identified *ADAMTS18* as the target gene for endothelial specific SE12313 and revealed a role for *ADAMTS18* in EC sprouting. At the same time, our work has led to the development of a lentiviral vector that specifically drives transgene expression in EC and is further regulated by hypoxia. Our strategy thus acts as a proof of principle for rational design of enhancer mediated cell-type specific gene therapy that are critical for the future of targeted treatments.

## DATA AVAILABILITY

RNA sequencing data obtained in this study are available from NCBI Gene Expression Omnibus (GEO) portal, accession number GSE151832. All publicly available next-generation sequencing data used in this study are listed in supplementary Table S4 with the references.

## Supplementary Material

gkab633_Supplemental_Files
