# Supplementary material for: Characterization of a functional endothelial super-enhancer that regulates ADAMTS18 and angiogenesis
Source: Nucleic Acids Res. 2021 Jul 28;49(14):8078–96. doi: 10.1093/nar/gkab633 (PMC8373076; doi:10.1093/nar/gkab633)
Supplement: gkab633_Supplemental_Files [file gkab633_supplemental_files.zip › gkab633_supplementary_data.pdf]

Characterization of a functional endothelial super-enhancer that regulates ADAMTS18 and angiogenesis

Isidore Mushimiyimana[1], Henri Niskanen[1], Mustafa Beter[1], Johanna P. Laakkonen[1], Minna U. Kaikkonen[1], Seppo Ylä-Herttuala[1,2†], Nihay Laham-Karam[1*†]

1) *A. I. Virtanen Institute for Molecular Sciences; University of Eastern Finland; Kuopio, 70211; Finland.*
2) *Heart Center and Gene Therapy Unit; Kuopio University Hospital; Kuopio, 70029; Finland*

[†]The authors wish it to be known that, in their opinion, the last two authors should be regarded as Joint Last Authors.

*correspondence: nihay.laham-karam@uef.fi

Supplementary Figures and Tables

Supplementary Figure 1

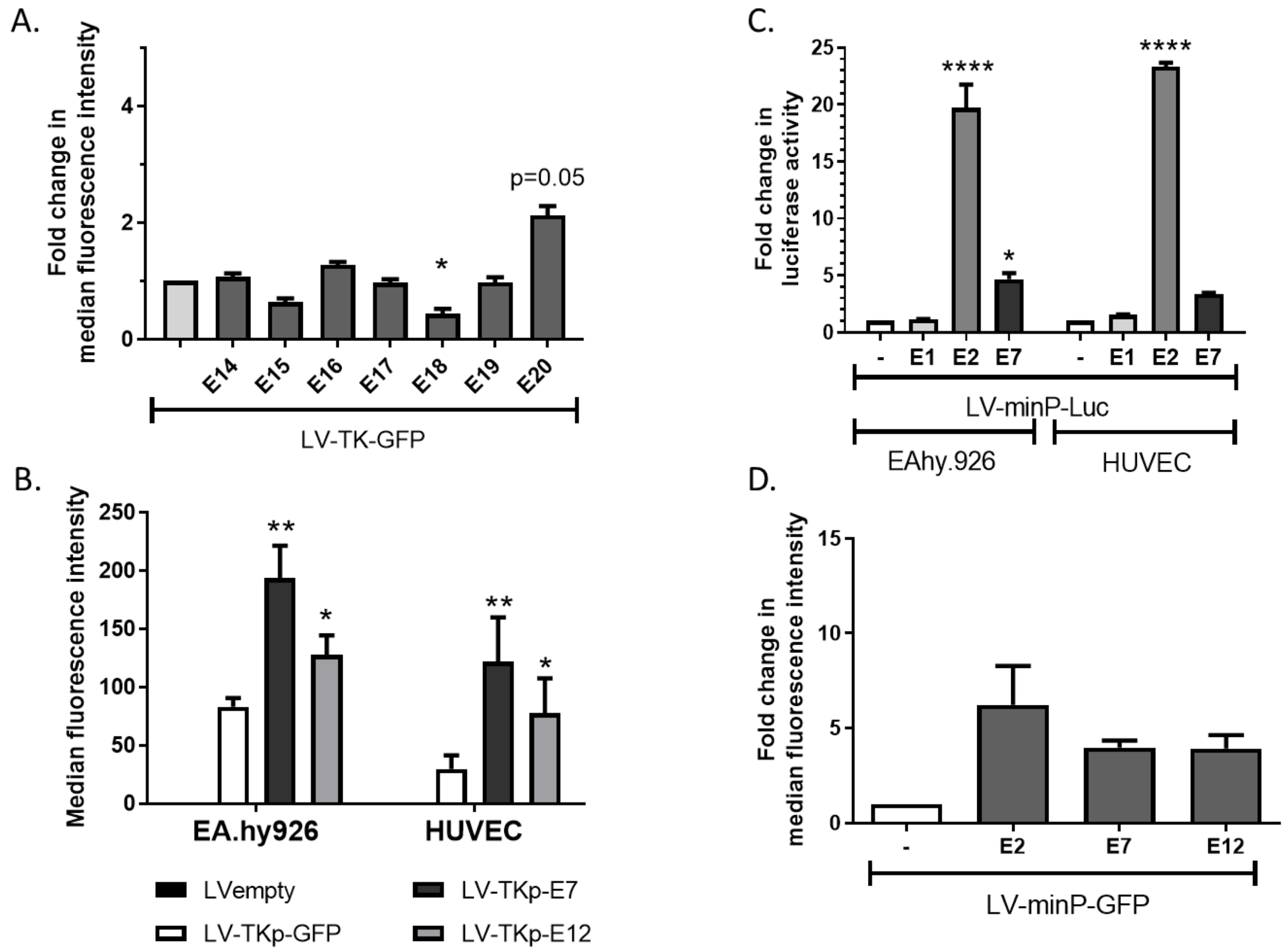

Supplementary Figure 1. *Enhancers are active in the context of TK promoter and with luciferase reported.* **A**. Flow-cytometric analysis of GFP expression in HUVEC transduced (d3) with control LV-TK-GFP or LV-TK-GFP-E(X) expressing different enhancers. **B.** HUVEC were transduced with LV-TK-GFP encoding E7 or E12 enhancers and GFP fluorescence quantitated after 3 days. **C.** HUVEC and EA.hy926 were transduced with LV-minP-Luc-E1, E2, E7 or the control vector and incubated for 72 hrs prior to reporter assay. Luciferase signal was measured with CLARIOstar microplate reader and normalised to control transductions activity. **D**. U87 glioblastoma cells were transduced with LV-minP-GFP-E2, E7 or E12 and GFP expression quantified. Fold change of median fluorescence intensity (MFI) was calculated relative to LV-minP-GFP transduced cells. Data is presented mean±SEM (n=3) and was analysed using ANOVA with Tukey's multiple comparison * p<0.05, ** p<0.01.

Supplementary Figure 2

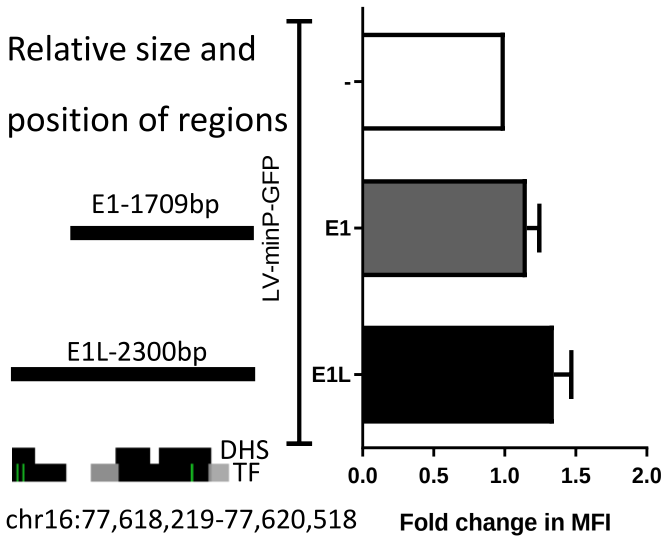

Supplementary Figure 2. *Effects of enhancer size on activity in vectors*. The activity of E1 and a larger fragment of E1 that included an additional DNase I HS sites (E1L) were tested in the context of LV-minP-GFP and by transduction of HUVEC. The regulatory activity of these regions on GFP expression was determined by flow cytometry. Fold change in median fluorescence activity was calculated compared to LV-minP-GFP transduced cells. Graphed data is presented as mean±SEM (n=3).

Supplementary Figure 3

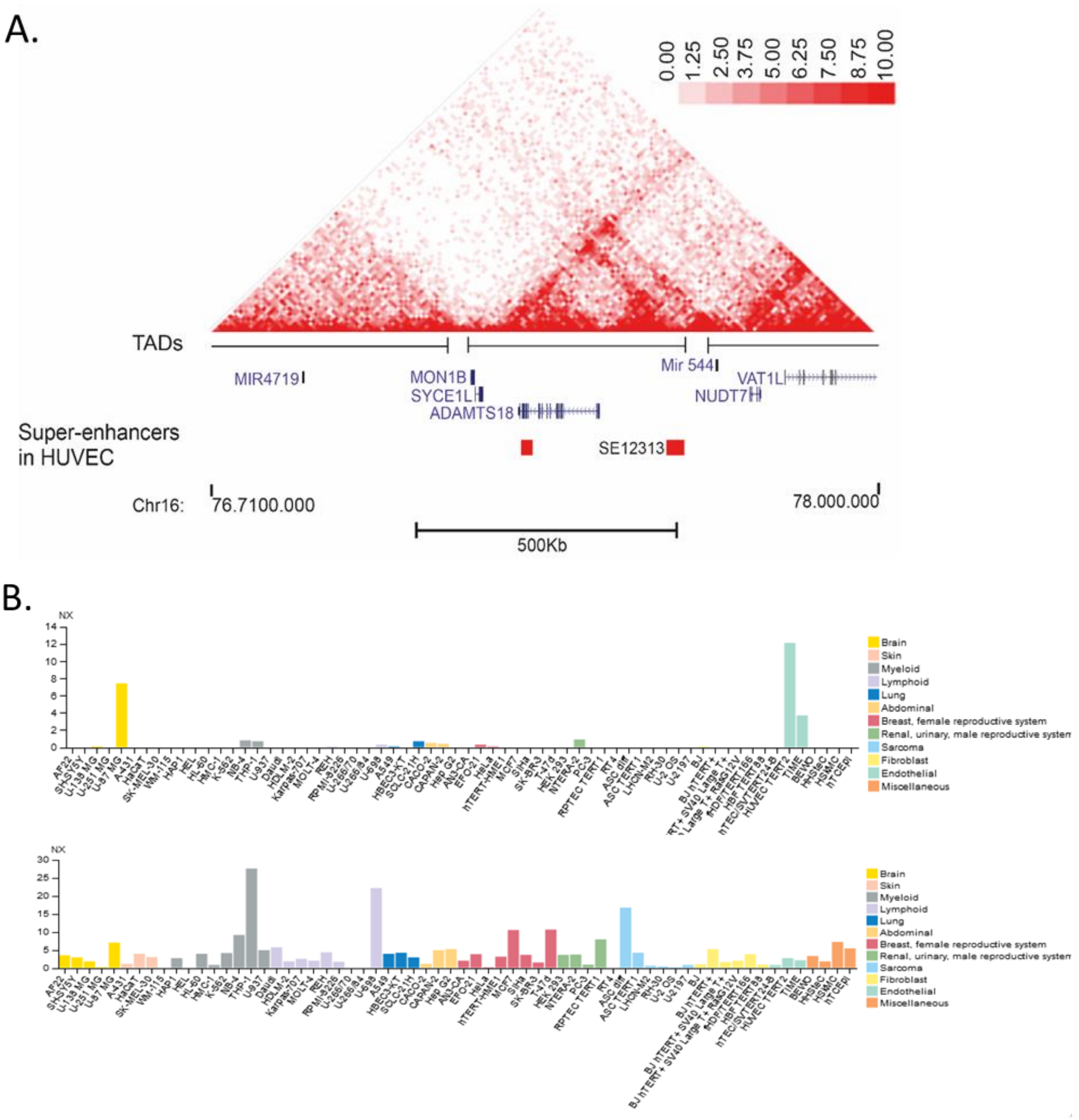

Supplementary Figure 3. A. *Hi-C demonstrating topological domains***.** Hi-C interactions are demonstrated along with deduced topological active domain (TAD) for the genome area that includes SE12313 and the surrounding genes. **B**. *RNA expression of ADMTS18 and NUDT7 in different cell lines.* RNA expression data and images from the Human Protein Atlas [http://www.proteinatlas.org; (53)] available from v19.3.proteinatlas.org for *ADAMTS18* (https://www.proteinatlas.org/ENSG00000140873-ADAMTS18/cell) and *NUDT7* (https://www.proteinatlas.org/ENSG00000140876-NUDT7/cell).

Supplementary Figure 4

A.

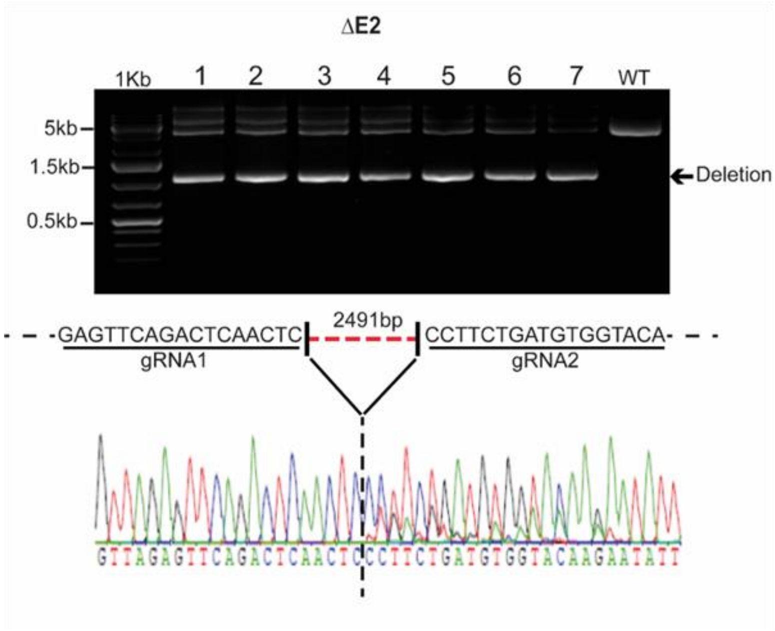

B.

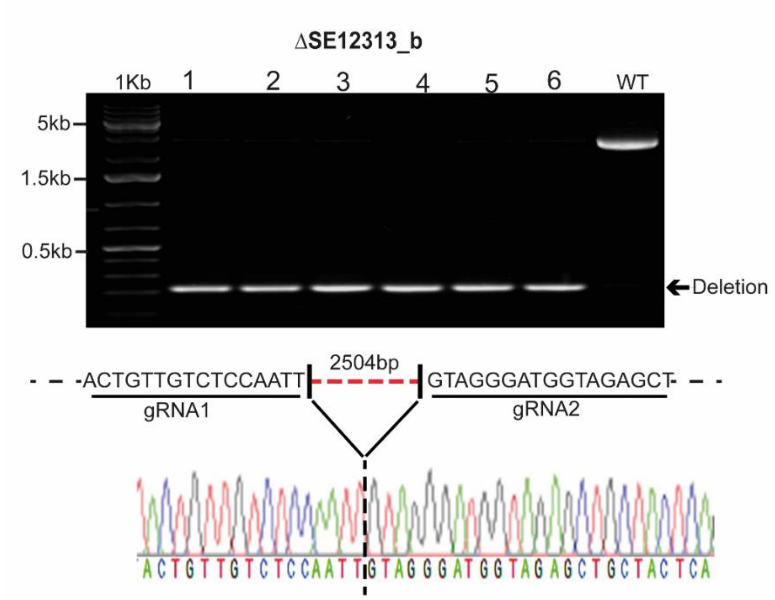

Supplementary Figure 4. *Detection of the enhancer deletions.* Confirmation of E2 (A) and SE12313-b (B) deletions in EA.hy926 cells by PCR and Sanger sequencing. Clone numbers are indicated on top of the gels, and genomic excision points of the gRNAs are indicated by the dashed lines in the Sanger sequencing panels.

Supplementary Figure 5

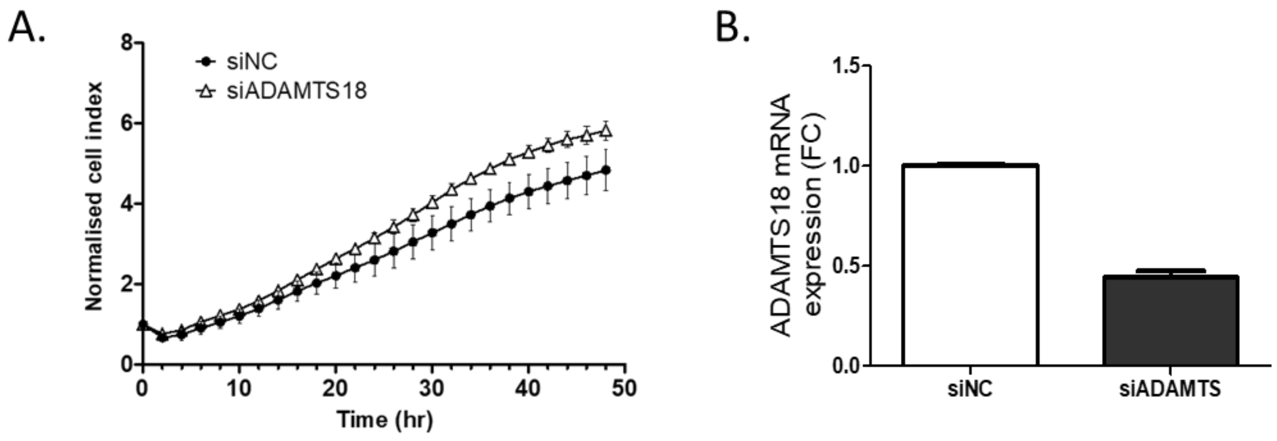

Supplementary Figure 5. *Knockdown of ADAMTS18 in EC.* A. Cell proliferation of EA.hy926, comparison of siNC and siADAMTS18 treated cells. Cell proliferation was determined using the XCelligence DP system for the indicated time (mean±SEM, n=3). D. DsiRNA2 knockdown of *ADAMTS18* mRNA in HUVEC as compared to controls DsiRNA (siNC; 2d after transfection; RT-qPCR data normalised to *ACTB* mRNA; mean±SEM, n=7).

Supplementary Table 1- List of primers used to amplify enhancer or promoter regions for cloning or genotyping.

Enhancer	Primer Name	Primer sequence 5' to 3'
	**primers for enhancer cloning**	
**E1**	XhoI Chr16:77584912-1 forw	AACTCGAGCTAAGCCAAGCGACAAGA
	ClaI Chr16:77586621-1 Rev	TTATCGATTTCATGCCCCCTCAAACCC
**E2**	XhoI Chr16:77593970-2 forw	AACTCGAGCATCAGGTTAGAGTTCAGACTCA
	ClaI Chr16:77596567-2 Rev	CAATCGATCCCAGCCACACTGTAACTTTATC
**E3**	XhoI Chr1:94091477-3 forw	AACTCGAGTTTCTGTCAAAGAACCGGAAGA
	ClaI Chr1:94093116-3 Rev	TAATCGATATCCAATGGCGTGAGGACCA
**E4**	XhoI Chr8:68008290-4 forw	AACTCGAGCCCAGCACTAACCGTTTT
	ClaI Chr8:68009949-4 Rev	TAATCGATATGGACCAGAAGCACACAGG
**E5**	XhoI Chr1:154484256-5 forw	AACTCGAGACACAGCGCTTGTCTGATTC
	ClaI Chr1:154485846-5 Rev	TTATCGATGGGGTGTGCATTGCAGAAAAT
**E6**	XhoI Chr18:71036310-6 forw	AACTCGAGAACTCTTCAAGCTGCCACC
	ClaI Chr18:71037918-6 Rev	TAATCGATCCCACAGACTACCCTACCCT
**E7**	XhoI Chr5:163759097-7 forw	AACTCGAGCAGCTTCACCTAAGGCCAAA
	ClaI Chr5:163761191-7 Rev	TAATCGATGGTTATCTACATGTCCCAGAGAGT
**E8**	XhoI Chr15:38827479-8 forw	AACTCGAGCTGCTCCACTGGGGCTTAT
	ClaI Chr15:38829657-8 Rev	ATATCGATTCAATGACTGGTCAAAGGGAGAA
**E9**	XhoI Chr2:190970508-9 forw	AACTCGAGATGCATGATGCCCTTCAGAGT
	ClaI Chr2:190972438-9 Rev	TTATCGATGTCCAAGAGAGCCAGAGCAC
**E10**	SalI Chr2:190979911-10 forw	AAGTCGACAATGACTTACCATGGCCCCTC
	ClaI Chr2:190981148-10 Rev	TTATCGATTCTCTAGGAGGCCCAGCAG
**E11**	ClaI Chr13:28355829-11 forw	TAATCGATCGAACAGAAGGGTGGTCGT
	ClaI Chr13:28357811-11 Rev	AAATCGATCAGGTTCACTAGAGCTCGGAT
**E12**	XhoI Chr13:28392221-12 forw	AACTCGAGCAGTGGCTGAGGAGAGAGGA
	ClaI Chr13:28394870-12 Rev	AAATCGATACCTAGGGAAGAGCCAACCT
**E13**	XhoI chr5:172175192-13 Forw	TTCTCGAGTCCTGGTATCTGGGGAGAGC
	ClaI chr5:172175192-13 Rev	TAATCGATGCCAGGTTGTCAGGAGGATG
**E14**	XhoI-SuEnh1049 forw	TACTCGAGAAAGGCTTGGCCCAGTA
	SalI-SuEnh1049 rev	ATAGTCGACCCCTGACACACAGCAAT
**E15**	XhoI-SuEnh4695 forw	ATCTCGAGTCCCTCCCCCAAGTTCAGTA
	SalI-SuEnh4695 rev	ATAGTCGACGGAGGAAGCCATCCTACATT
**E16**	XhoI-SuEnh9678 forw	ATCTCGAGTGAATATGATTCTTGCCTTGAGA
	SalI-SuEnh9678 rev	ATAGTCGACAACTAAACTAAACTCTTTTTCCCC
**E17**	XhoI-SuEnh9698 forw	TACTCGAGGGAGGGAGGAGAAGAAGC
	SalI-SuEnh9698 rev	TAAGTCGACAGGCATAACTGCTGTGTCC
**E18**	XhoI-SuEnh29577 forw	ATCTCGAGTCAAACCAGTAGAGACAGCAG
	SalI-SuEnh29577 rev	AATGTCGACTGCTGGAGCCCAAAATC
**E19**	XhoI-SuEnh30727 forw	TACTCGAGCTTTGTCCTGAGACGGG
	SalI-SuEnh30727 rev	ATTGTCGACGTTCACTCCGAGTTTCT
**E20**	XhoI-SuEnh17789 forw	ATCTCGAGTCCTCTGTCAATGCCAC
	SalI-SuEnh17789 rev	ATAGTCGACGGATCTAAAGTGTGTCTCCTG

Supplementary Table 1 cont.

Enhancer	Primer Name	Primer sequence 5' to 3'
**Primers for all enhancers from SE12313 and 26147**		
**SE12313-a**	XhoISE12313-1 Forw	CTCGAGCACATTGGGTCTCTCAAGCAGG
	ClaISE12313-1 Rev	ATCGATAGAAGGAGTGACCTAACAGAGGACAGG
**SE12313-b**	SalISE12313-2 Forw	GTCGACAGCCCAGGAGGGAGACAAGG
	ClaISE12313-2 Rev	ATCGATACATGGATGATGGGAGAGCTGAG
**SE12313-c**	XhoISE12313-3 forw	CTCGAGCCTATGCAACAAACACGCATGTTC
	SalISE12313-3 Rev	GTCGACGGATACATACTGAAAATTGTATAGTACGG
**SE26417-a**	XhoI-SE7-1 forw	CTCGAGCTAACTGGGCCACAAAGG
	ClaI-SE7-1 rev	ATCGATTGACCCCCAGACTACCCAT
**SE26417-c**	XhoI-SE7-2 forw	CTCGAGTCCCCTGCATCCTCTGTCAAC
	ClaI-SE7-2 rev	ATCGATAAGAAGTAAGGCTTCTCTCCAGT
**SE26417-d**	XhoI-SE7-3 forw	CTCGAGACGTGAAACAGCAGTGACAG
	ClaI-SE7-3 rev	ATCGATGGGAATCAGAGATGCATAATGAAA
**primers for E2 size variants**		
**E2(875)**	XhoI SE2-DHS1.5-forw	TTCTCGAGTCCATATCCACAGATTGCCCAC
	SalI-SE2-DHS1.5-rev	ATTGTCGACTTGCTGTTGTGCAACTTTTCCAT
**E2(1284)**	XhoI-SE2-DHS2-forw	TACTCGAGCAGTTCACCTTCCATTGCATGAC
**Primers for longer E1**		
**E1L**	SalI-SE1L forw	GTCGACATTCAGTACAGTGCCAAGCG
	ClaI-SE1L rev	ATCGATGTACTGTGGTCCCAACGC
**Primers for SE12313 enhancer deletions**		
**ΔSE12313_b**	Chr16_SEdel Forw	TGGCCTTCCTACTTGCACTC
	Chr16_SEdel CTL Rev	GGGGAAGAGCAAGGGCTAAA
**ΔE2**	P560_SE2_forw	GGTTGCCTGATTCCTGAATAGC
	SE2L-Rev	ATGTGCAAGATGGCTCCCT
**Primers for promoter cloning**		
**ADAMTS 18p**	AgeI ADAMTS18p Rev	TTACCGGTCAGGTGCGGACGC
	EcoRV ADAMTS18p Forw	TTGATATCGGTGTTGAGGACGCTATCTTG
**NUDT7p**	AgeI NUDT7 Rev	TACCGGTGCCCTGGGGAATGTTTG
	EcoRV NUDT7 Forw	AGATATCAAGCGATCCTCCCACCTCAGC
**minP**	minP_linker	ATCGATACACTAGAGGGTATATAATGGAAGCTCGACTTCCAGCTTACCGG

Supplementary Table 2- List of crRNAs used in CRISPR deletion of enhancers.

Deleted region	crRNA name	crRNA sequence	Deletion size(bp)
**SE12313-b**	SE12313-b left	AAAGAAGCTTCCGGTTGAAT	2504
	SE12313-b right	CTATTATTTCTCCCGACGTA	
**E2**	E2 left	TGTACCACATCAGAAGGCTT	2491
	E2 right	GAGTTCAGACTCAACTCTTG	

Supplementary Table 3- List of qPCR assays used.

Gene target	qPCR assay ID	Supplier
ADAMTS18	HsPT581326160	IDTDNA
VEGFA	Hs00900055_m1	IDTDNA
IGFN1	HsPT5839776488	IDTDNA
TRIPM	HsPT5827084105	IDTDNA
RIBC2	HsPT582330025	IDTDNA
ACTB	4333762T	Applied Biosystems

Supplementary Table 4- List of all genome-wide sequencing data used in this study

Cell and library type	Reference	Gene Expression Omnibus Database ID
HUVEC GRO-seq	Niskanen et al (41)	GSE94872
RWPE Hi-C	Rickman et al (84)	GSM927076
hESC Hi-C	Dixon et al (85)	GSM862723
IMR90 Hi-C	Dixon et al (85)	GSM862724
HUVEC Hi-C	Niskanen et al (41)	GSE94872
HUVEC ETS1 ChIP-Seq	Wang et al (86)	GSM2947437
HUVEC ERG ChIP-Seq	Kalna et al (48)	GSM3557980
HUVEC FL1 ChIP-Seq	Wang et al (86)	GSM2947445
HUVEC RBPJ ChIP-Seq	Wang et al (86)	GSM2947453
HUVEC GATA2 ChIP-Seq	Wang et al (86)	GSM2947457
HUVEC EP300 ChIP-Seq	Wang et al (86)	GSM2947461
HUVEC EZH2 ChIP-Seq	Wang et al (86)	GSM2947465
HUVEC NFATc1 ChIP-Seq	Suehiro et al (87)	GSE49429
HUVEC p300 ChIP-Seq	Zhang et al (88)	GSE41166
HUVEC MEF2C ChIP-Seq	Maejima et al (89)	GSE32644
HUVEC JUN ChIP-Seq	ENCODE Cons. (90)	GSE31477, GSM935278
HUVEC cFOS ChIP-Seq	ENCODE Cons. (90)	GSM730703, GSM730704
HUVEC cMyc ChIP-Seq	ENCODE Cons. (90)	GSM748547
HUVEC CTCF ChIP-Seq	ENCODE Cons. (90)	GSM749674
HUVEC HIF1α ChIP-Seq	Mimura at al (91)	GSM955978
EAhy ΔE2_1 RNA-Seq	This study	GSE151832
EAhy ΔSE12313_b_1 RNA-Seq	This study	GSE151832
EAhy_CTRL RNA-Seq	This study	GSE151832

Supplementary Table 5- Cell cycle results for individual clones of ΔE2 and Δ SE12313-b (mean±SEM, n=4).

	**EA.hy926**	**ΔE2**			**ΔSE12313_b**		
	**CTL**	#1	#4	#5	#1	#4	#5
**G0/G1 (%)**	60.93±2.5	64.03±2.4	64.13±1.5	64.00±1.9	64.58±1.65	64.34±1.36	64.89±2.37
**S (%)**	23.82±1.0	20.53±1.3	19.92±0.3	20.71±0.6	20.28±0.56	20.08±0.64	19.85±1.29
**G2/M (%)**	11.42±1.3	10.99±1.3	11.20±1.12	9.54±1.3	9.80±0.83	10.76±1.15	9.41±1.1

Supplementary Table 6- GSEA identified genes among DEGs in ΔE2 and ΔSE12313-b that are associated with negative regulation of cell cycle

**Gene**	**GO term**	**GO accession number**	**Biological process included gene**
AURKA	negative regulation of G2/M transition of mitotic cell cycle	GO:0010972	Mitotic nuclear division & Cell division
BLM	negative regulation of cell division	GO:0051782	DNA replication
CCNF	negative regulation of centrosome duplication	GO:0010826	Mitotic nuclear division & Cell division
CDT1	negative regulation of protein localization to kinetochore	GO:1905341	DNA replication & G1/S transition
CHEK1	negative regulation of mitotic nuclear division	GO:0045839	DNA replication
WEE1	negative regulation of G1/S transition of mitotic cell cycle	GO:2000134	Mitotic nuclear division & Cell division
MAD2L1	negative regulation of mitotic cell cycle	GO:0045930	Cell division & Sister chromatid cohesion
RAD21	negative regulation of mitotic metaphase/anaphase transition	GO:0045841	sister chromatid cohesion & cell division
RRM2	negative regulation of G0 to G1 transition	GO:0070317	DNA replication

References

84. Rickman,D.S., Soong,T.D., Moss,B., Mosquera,J.M., Dlabal,J., Terry,S., MacDonald,T.Y., Tripodi,J., Bunting,K., Najfeld,V., et al. (2012) Oncogene-mediated alterations in chromatin conformation. *Proc. Natl. Acad. Sci. U. S. A.,* **109,** 9083-9088.

85. Dixon,J.R., Selvaraj,S., Yue,F., Kim,A., Li,Y., Shen,Y., Hu,M., Liu,J.S. and Ren,B. (2012) Topological domains in mammalian genomes identified by analysis of chromatin interactions. *Nature,* **485,** 376-380.

86. Wang,S., Chen,J., Garcia,S.P., Liang,X., Zhang,F., Yan,P., Yu,H., Wei,W., Li,Z., Wang,J., et al. (2019) A dynamic and integrated epigenetic program at distal regions orchestrates transcriptional responses to VEGFA. *Genome Res.,* **29,** 193-207.

87. Suehiro,J., Kanki,Y., Makihara,C., Schadler,K., Miura,M., Manabe,Y., Aburatani,H., Kodama,T. and Minami,T. (2014) Genome-wide approaches reveal functional vascular endothelial growth factor (VEGF)-inducible nuclear factor of activated T cells (NFAT) c1 binding to angiogenesis-related genes in the endothelium. *J. Biol. Chem.,* **289,** 29044-29059.

88. Zhang,B., Day,D.S., Ho,J.W., Song,L., Cao,J., Christodoulou,D., Seidman,J.G., Crawford,G.E., Park,P.J. and Pu,W.T. (2013) A dynamic H3K27ac signature identifies VEGFA-stimulated endothelial enhancers and requires EP300 activity. *Genome Res.,* **23,** 917-927.

89. Maejima,T., Inoue,T., Kanki,Y., Kohro,T., Li,G., Ohta,Y., Kimura,H., Kobayashi,M., Taguchi,A., Tsutsumi,S., et al. (2014) Direct evidence for pitavastatin induced chromatin structure change in the KLF4 gene in endothelial cells. *PLoS One,* **9,** e96005.

90. ENCODE Project Consortium. (2012) An integrated encyclopedia of DNA elements in the human genome. *Nature,* **489,** 57-74.

91. Mimura,I., Nangaku,M., Kanki,Y., Tsutsumi,S., Inoue,T., Kohro,T., Yamamoto,S., Fujita,T., Shimamura,T., Suehiro,J., et al. (2012) Dynamic change of chromatin conformation in response to hypoxia enhances the expression of GLUT3 (SLC2A3) by cooperative interaction of hypoxia-inducible factor 1 and KDM3A. *Mol. Cell. Biol.,* **32,** 3018-3032.
